# RNA-Seq Reveals Different Gene Expression in Liver-Specific Prohibitin 1 Knock-Out Mice

**DOI:** 10.3389/fphys.2021.717911

**Published:** 2021-09-03

**Authors:** Kyuwon Lee, Hyeonju Yu, Stephanie Shouse, Byungwhi Kong, Jihye Lee, Seong-Ho Lee, Kwang Suk Ko

**Affiliations:** ^1^Department of Nutritional Science and Food Management, College of Science and Industry Convergence, Ewha Womans University, Seoul, South Korea; ^2^Center of Excellence for Poultry Science, University of Arkansas System Division of Agriculture, Fayetteville, AR, United States; ^3^Department of Nutrition and Food Science, College of Agriculture and Natural Resources, University of Maryland, College Park, MD, United States; ^4^Karsh Division of Gastroenterology and Hepatology, Department of Medicine, Cedars-Sinai Medical Center, Beverly Hills, CA, United States

**Keywords:** prohibitin 1, RNA-seq, gene expression, liver disease, hepatotoxicity

## Abstract

Prohibitin 1 (PHB1) is an evolutionarily conserved and ubiquitously expressed protein that stabilizes mitochondrial chaperone. Our previous studies showed that liver-specific Phb1 deficiency induced liver injuries and aggravated lipopolysaccharide (LPS)-induced innate immune responses. In this study, we performed RNA-sequencing (RNA-seq) analysis with liver tissues to investigate global gene expression among liver-specific Phb1^−/−^, Phb1^+/−^, and WT mice, focusing on the differentially expressed (DE) genes between Phb1^+/−^ and WT. When 78 DE genes were analyzed for biological functions, using ingenuity pathway analysis (IPA) tool, lipid metabolism-related genes, including insulin receptor (Insr), sterol regulatory element-binding transcription factor 1 (Srebf1), Srebf2, and SREBP cleavage-activating protein (Scap) appeared to be downregulated in liver-specific Phb1^+/−^ compared with WT. Diseases and biofunctions analyses conducted by IPA verified that hepatic system diseases, including liver fibrosis, liver hyperplasia/hyperproliferation, and liver necrosis/cell death, which may be caused by hepatotoxicity, were highly associated with liver-specific Phb1 deficiency in mice. Interestingly, of liver disease-related 5 DE genes between Phb1^+/−^ and WT, the mRNA expressions of forkhead box M1 (Foxm1) and TIMP inhibitor of metalloproteinase (Timp1) were matched with validation for RNA-seq in liver tissues and AML12 cells transfected with *Phb1* siRNA. The results in this study provide additional insights into molecular mechanisms responsible for increasing susceptibility of liver injuries associated with hepatic Phb1.

## Introduction

Liver disease is a comprehensive term, including diverse stages of the disorder related to liver injury, and is one of the main causes of illness and death worldwide. According to Globocan 2018 by the WHO, the incidence and mortality of liver cancer are the sixth and third highest in both sexes and all ages, respectively (Bray et al., 2018). Although the risk factors include hepatitis B and C virus, alcohol, obesity, etc., the cellular regulatory mechanisms for liver disease and accountable evidence for physiological responses are yet to be explored (Smalling et al., 2013). It may be crucial to identify specific genes and pathway changes by liver injuries through whole transcriptome analyses of a relevant animal model. RNA-sequencing (RNA-seq) can proceed with a whole-genome survey of gene expression and provide a digital measure of the presence and prevalence of transcripts from known and previously unknown genes (Wang et al., 2010; Benjamin et al., 2014). The objective of the present study was to conduct global gene expression analyses of liver-specific Prohibitin 1 (Phb1) knockout (KO) mice as a liver disease model. PHB1 is an evolutionarily conserved and ubiquitously expressed protein that stabilizes mitochondrial chaperone (Yang et al., 2018). The protein is located to inner mitochondrial membrane and nucleus, so it regulates several important cellular processes, including apoptosis, cell proliferation, and transcriptional regulation by interacting with retinoblastoma protein (Rb), p53, and E2F transcription factors (Nijtmans et al., 2000; Ramani et al., 2016).

Liver-specific Phb1 homozygous KO (referred to as Phb1^−/−^) mouse showed positive staining for OV-6, an oval cell marker, and glutathione S-transferase Pi (GST Pi), a preneoplastic marker, at 3 weeks old and exhibited spontaneous liver injury, fibrosis, and hepatocellular carcinoma (HCC) from 8 months of age (Ko et al., 2010). A follow-up research study demonstrated that lipopolysaccharide (LPS)-innate immune responses were exacerbated by Phb1 deficiency, which mimics Phb1^−/−^, in murine macrophages (Jung et al., 2020). Furthermore, another research showed that there were no significant differences in body weight between liver-specific Phb1 heterozygote (referred to as Phb1^+/−^) and wild-type (WT, referred to as Phb1^+/+^) mice, but Phb1^+/−^ showed more severe liver damage when feeding a methionine or choline-deficient diet, compared with WT (Heo and Ko, 2019).

Collectively, these results suggested that liver-specific Phb1 deficient mice may be a suitable model to investigate molecular mechanisms related to hepatotoxicity. In an earlier study, we observed phenotypic differences and differential gene expression, using microarray assay between Phb1^−/−^ and WT (Ko et al., 2010). On the basis of the result, we have also investigated the vulnerability of hepatotoxicity in deletion of Phb1 (Heo and Ko, 2019; Jung et al., 2020). As part of research elucidating the susceptibility, it is necessary to observe the genetic change in liver-specific Phb1^+/−^, compared with WT to determine which genetic factors increases the susceptibility of liver injuries. In the current study, we aimed to investigate genome-wide gene expression among liver-specific Phb1^−/−^, Phb1^+/−^, and WT mice by using the RNA-seq method, focusing on the differentially expressed (DE) genes between Phb1^+/−^ and WT. Also, we verified the expression level of hepatic disease-related genes in normal murine hepatocytes transfected with *Phb1* siRNA to compare RNA-seq data. Through this comparison, DE genes showing greater differences between Phb1^+/−^ and WT may provide sharper perceptions of alteration of physiological responses in a liver-specific Phb1-deficient mouse as a liver disease model.

## Materials and Methods

### Animals, Liver Tissue, and Total RNA Isolation

This study about sample collection was approved by Cedars-Sinai Medical Centers (Los Angeles, CA, USA) (IACUC 9370). Liver-specific Phb1 deficient mice were generated as previously described (Ko et al., 2010). Briefly, liver-specific Phb1 KO [*Phb1*^*loxp*/*loxp*^, *Albumin-Cre*^+/+^ (*Alb-Cre*^+/+^)] mice on a C57BL/6 background, and *Alb-Cre*^+/+^ mice were maintained for the generation of liver-specific Phb1-deficient mice. Selected three males and three females per Phb1^−/−^, Phb1^+/−^, and WT (4–5 weeks old, *n* = 6/genotype) were sacrificed for the harvest of liver tissue. Obtained samples were stored at −80°C until analysis. Total RNA was isolated from liver tissue, using TRIzol reagent (ThermoFisher, Waltham, MA, USA) according to the guideline of the manufacturer.

### RNA-seq and Data Analysis

The RNA quality based on the RNA integrity number (RIN) was assessed using the RNA R6K assay for the Agilent 2200 TapeStation (Agilent Technology, Santa Clara, CA, USA). RIN scores were ranged between 8.5 and 9.5. For the RNA-seq analysis, library preparation and sequencing analyses were carried out at the Research Technology Support Facility of Michigan State University (East Lansing, MI, USA). Transcriptome (*n* = 6 for each WT, Phb1^+/−^, and Phb1^−/−^ mice, including three males and three females per group) was analyzed, using a 1 × 50 bp single-end-read method of Illumina HiSeq system (Illumina Inc., San Diego, CA, USA) as described in earlier reports (Kong et al., 2017; Lee et al., 2020). The raw reads were aligned with the mouse reference genome (GRCm38.p6) downloaded from NCBI (https://www.ncbi.nlm.nih.gov/assembly/GCF_000001635.26) using ArrayStar program in Lagergene software package (DNAStar Inc., Madison, WI, USA). Total mapped counts were transformed into log_2_ values of the number of reads per million (RPM) to stabilize the variance, and then further quantile normalization was performed. Normalized values were subjected to further statistical analyses performed by JMP Genomics 9 (SAS Institute Inc., Cary, NC). The one-way ANOVA statistics was used to compare among WT, Phb1^+/−^, and Phb1^−/−^ mice. Genes showing >2-fold changes (1 in log_2_) differences and <0.05 false discovery rate (FDR) in the comparisons were considered DE genes. The raw data were submitted to the National Center for Biotechnology Information (NCBI) Sequence Read Archive (SRA) database under accession numbers: SRR14876823, SRR14876822, SRR14876815, SRR14876812, SRR14876818, SRR14876817, SRR14876813, SRR14876821, SRR14876820, SRR14876819, SRR14876816, SRR14876814, SRR14876807, SRR14876811, SRR14876810, SRR14876809, SRR14876808, and SRR14876806.

### Bioinformatics Pathway Analyses

For pathway analyses, ingenuity pathway analysis (IPA; Qiagen, Valencia, CA; http://www.ingenuity.com) software was used for functional annotation, canonical pathway analysis, upstream analysis, and network discovery. The IPA core analyses are based on previous knowledge of the associations of upstream regulators and their downstream target genes archived in the ingenuity knowledge base. The *p*-values were calculated by Fisher's exact test for the upstream regulator analysis.

### Validation for RNA-seq Using RT-PCR in Liver Tissues

For cDNA synthesis and PCR, a Verso cDNA synthesis kit (ThermoFisher, Waltham, MA, USA) and Promega PCR master mix (Promega Corp., Madison, WI, USA) were used. PCR reactions were carried out at 95°C for 2 min, followed by 22 or 32 cycles with denaturation at 95°C for 30 s and annealing and elongation at 55°C for 45 s and 72°C for 1 min. Band density was determined, using Image J software (National Institutes of Health, Bethesda, MD, USA). The gene expression levels were normalized to the expression levels of the GAPDH housekeeping gene. Relative quantification was reported as fold changes compared to control samples. The primer sequence of RT-PCR used in the experiment is shown in Supplementary Material 1.

### Cell Culture

The normal murine hepatocyte cell line, AML12 cells were obtained from American Type Culture Collection (ATCC, Manassas, VA, USA). AML12 cells were cultured in a 1:1 mixture of Dulbecco's modified Eagle's medium and Ham's F12 medium (Cytiva/Hyclone, Marlborough, MA, USA), supplemented with 10% fetal bovine serum (FBS) (ThermoFisher, Waltham, MA, USA), 1% penicillin and streptomycin (ThermoFisher, Waltham, MA, USA), 0.5 mM sodium pyruvate (Sigma-Aldrich, St. Louis, MO, USA), 5 μg/ml insulin, 5 μg/ml transferrin, and 5 ng/ml selenium (ITS) (Sigma-Aldrich, St. Louis, MO, USA), 40 ng/ml dexamethasone (Sigma-Aldrich, St. Louis, MO, USA), and 0.6 g of sodium bicarbonate (Daejung Co., Siheung, Korea). The cells were maintained at 37°C and in a 5% CO_2_ humidified incubator.

### Knockdown of Prohibitin 1 by Small Interfering RNAs

Predesigned small interfering RNA (siRNA) targeting mouse *Phb1* (ThermoFisher, Waltham, MA, USA) with different knockdown (KD) efficiency and nonspecific scrambled siRNA (ThermoFisher, Waltham, MA, USA) were purchased. Briefly, AML12 cells were seeded and transfected in a six-well plates at density of 0.2 × 10^6^ cells/well with 13 nM si*Phb1* (sense: AGAGCGAGCGGCAACAUUTT, antisense: AAAUGUUGCCGCUCGCUCUGT), which mimics Phb1^−/−^, 50-nM si*Phb1* (sense: GCCGCUGUCAAUAAAUCACTT, antisense: GUCAUUUAUUGACAGCGGCTT), which mimics Phb1^+/−^, or scramble siRNA supplemented with Lipofectacmine RNAi MAX (ThermoFisher, Waltham, MA, USA), following the guideline of the manufacturer.

### qRT-PCR for Expression of Target Genes

Total RNA was isolated from AML12 cells transfected with siRNAs by using TRIzol reagent (ThermoFisher, Waltham, MA, USA) according to the guideline of the manufacturer. First-strand complementary DNA (cDNA) was prepared from 2 μg of total RNA, using RevertAid First Strand cDNA Synthesis Kit (ThermoFisher, Waltham, MA, USA) according to manufacturer's protocol. Quantitative PCR (qPCR) was performed with the Maxima SYBR Green/ROX qPCR Master Mix (2X) (ThermoFisher, Waltham, MA, USA). The reaction was performed in a volume of 10 μl, containing 50 ng of cDNA, 0.3 μM of each primer (Supplementary Material 2) and 5μl maxima SYBR Green/ROX qPCR Master Mix (2X), which were run in duplicate, using a QuantStudio3 thermocycler (ThermoFisher, Waltham, MA, USA). Amplification was carried out with a template denaturation at 95°C for 10 min, followed at 95°C for 15 s and 60°C for 1 min for 40 cycles. The gene expression levels were normalized to the expression levels of the β-actin housekeeping gene and calculated using the 2^−ΔΔCt^ method.

### Statistical Analysis

Data on validation using RT-PCR and qRT-PCR were presented as the mean ± standard deviation. Differences among groups were determined by one-way ANOVA analysis with Duncan *post-hoc* test, using SAS 9.4 (SAS Institute Inc., Cary, NC, USA). Values of *p* < 0.05 were considered statistically significant.

## Results

### RNA-seq Results

A total of 18 RNA-seq libraries were constructed using RNA samples extracted from the liver obtained from WT, Phb1^+/−^, and Phb1^−/−^. After filtering low read counts and normalization, a total of 8,982 transcripts remained. Of those, only mRNAs showing FDR <0.05 and log_2_ fold change >1 or <−1 (linear fold change >2 or <−2) were considered differentially expressed (DE) genes for further bioinformatics analyses and PCR validation. Using these criteria, this study was able to select 78 (15 up- and 63 downregulation) DE genes in Phb1^+/−^ mice, compared with WT. The complete gene list for 78DE genes is provided in Supplementary Material 3. Several genes, including optic atrophy 1 (Opa1), mitofusin 1 (Mfn1), and mitofusin 2 (Mfn2), which are involved in mitochondrial fusion and fission, contributing to mitochondrial morphology, were not differentially expressed between Phb1^+/−^ and WT. Expression of mitochondrial cytochrome c oxidase (Cox) responsible for aerobic energy generation was also no difference in Phb1^+/−^, compared with WT (data not shown). But these mitochondrial-related genes are downregulated in Phb1^−/−^ (data not shown). This result can indicate that half of the reduction in Phb1 expression may not affect mitochondrial dysfunction.

### The 10 Most DE Genes in Hetero Compared With WT

The full name of the 10 most DE genes in the Phb1^+/−^ group compared WT (plus FC values and FDR in KO compared with WT) and their functional characteristics are shown in Tables 1, 2. Of those, upregulated genes were involved in nucleolar functions (Nol3, Hist1h2b, and Snora17), xenobiotic enzyme (Sult1e1), ubiquitin peptidase (Usp2), and circadian functions (Ciart, Per2). The 10 most downregulated genes appeared to be involved in potential transcription factors (Mybl1), telomere length regulation (Rad51ap1), mitochondrial enzyme (Pycr1), DNA synthesis (Kifc1), receptors (Fzd3), coagulation factor (F13a1), TIMP inhibitor of metalloproteinase (Timp1), adapter protein in receptor tyrosine kinase (Shc4), membrane transporter (Abcc12), and structural protein (Tubb2b).

**Table 1:** The 20 most differentially expressed genes, comparing between Phb1^+/−^ vs. WT, Phb1^−/−^ vs. WT, and Phb1^−/−^ vs. Phb1^+/−^.

**Symbol**	**Entrez gene name**	**Hetero vs. WT**		**KO vs. WT**		**KO vs. Hetero**	
		**Log_**2**_FC**	**FDR**	**Log_**2**_FC**	**FDR**	**Log_**2**_FC**	**FDR**
Gm40498	Predicted gene, 40498	7.0	1.3E-02	10.0	1.0E-03	3.0	3.2E-01
HIST1H2BM	Histone cluster 1 H2B family member m	5.5	3.5E-02	2.3	4.2E-01	−3.2	2.3E-01
SULT1E1	Sulfotransferase family 1E member 1	2.7	3.8E-03	2.4	8.7E-03	−0.3	7.9E-01
CIART	Circadian associated repressor of transcription	1.4	4.0E-02	0.4	5.8E-01	−1.0	1.6E-01
Gm38832	Predicted gene, 38832	1.4	2.7E-04	−1.2	7.0E-04	−2.6	3.4E-07
Gm40368	Predicted gene, 40368	1.3	3.9E-02	2.5	3.5E-04	1.2	4.7E-02
USP2	Ubiquitin specific peptidase 2	1.3	9.4E-03	−0.6	2.4E-01	−1.8	5.2E-04
NOL3	Nucleolar protein 3	1.1	4.8E-02	4.2	3.5E-07	3.2	8.4E-06
Snora17	Small nucleolar RNA, H/ACA box 17	1.0	1.9E-02	2.2	4.0E-05	1.2	8.2E-03
PER2	Period circadian regulator 2	1.0	2.1E-02	0.6	1.9E-01	−0.4	3.6E-01
MYBL1	MYB proto-oncogene like 1	−3.3	2.1E-02	3.8	8.0E-03	7.1	4.1E-05
RAD51AP1	RAD51 associated protein 1	−3.5	2.3E-02	3.3	2.9E-02	6.8	1.4E-04
PYCR1	Pyrroline-5-carboxylate reductase 1	−3.8	9.0E-03	5.3	8.0E-04	9.1	3.9E-06
KIFC1	Kinesin family member C1	−3.9	4.8E-02	2.2	2.8E-01	6.0	3.2E-03
FZD3	Frizzled class receptor 3	−3.9	2.3E-02	5.3	3.5E-03	9.2	2.3E-05
F13A1	Coagulation factor XIII A chain	−4.1	1.1E-02	4.6	4.9E-03	8.7	1.7E-05
TIMP1	TIMP metallopeptidase inhibitor 1	−4.4	1.2E-02	6.4	6.9E-04	10.8	4.4E-06
SHC4	SHC adaptor protein 4	−4.5	7.3E-03	5.1	3.0E-03	9.7	8.9E-06
ABCC12	ATP binding cassette subfamily C member 12	−4.6	3.9E-03	3.9	1.3E-02	8.5	1.6E-05
TUBB2B	Tubulin beta 2B class IIb	−4.8	1.5E-02	7.6	5.2E-04	12.4	4.2E-06

“*Hetero” and “KO” in tables represent “Phb1^+/−^” and “Phb1^−/−^”, respectively*.

**Table 2:** Biological functions of the 10 most up- and down-regulated genes between Phb1^+/−^ vs. WT.

**Symbol**	**Functions**
Gm40498	• Predicted gene
Hist1h2bm	• Is a protein which is intronless and encodes a member of the histone H2B family
Sult1e1	• Is an estrogen sulfotransferase (EST), which is a major sulfotransferase isoform • Expresses in multiple human tissues • Regulates estrogen homeostasis by sulfonating and deactivating estrogen (Guo et al., 2015)
Ciart	• Forms a complex with other clock proteins and modulates the circadian machinery (Hatanaka and Takumi, 2017) • Associates mutant human CIART gene with grade 4 astrocytoma in human brain (Lee et al., 2017)
Gm38832	• Predicted gene
Gm40368	• Predicted gene
Usp2	• Is a member of the family of de-ubiquitinating enzymes, encoding a ubiquitin-specific protease • Stabilizes mouse double minute 2 (MDM2) and mouse double minute 4 (MDM4) to degrade p53 (Young et al., 2019) • Knockdown of USP2 inhibits hepatocyte apoptosis by elevating levels of c-Flip as an anti-apoptotic protein (Haimerl et al., 2009)
Nol3	• Is an apoptosis repressor with caspase recruitment domain • Can protect against cell death by interacting with Bax, preventing mitochondrial dysfunction (Gustafsson et al., 2004) • Ras induces NOL3 in epithelial cancers and NOL3 play a role in the oncogenic actions of Ras (Wu et al., 2010)
Snora17	• Is a non-coding small RNA; unknown function
Per2	• Is a member of the Period family of gene, which plays a role in circadian rhythms • Polymorphisms in this gene may increase the risk of getting certain cancers and have been linked to sleep disorders • Loss of the clock gene PER2 decreases erythrocyte life span (Sun et al., 2017)
Mybl1	• Expresses predominantly as a tissue-specific transcription factor in spermatocytes and breast epithelial cells (Tang and Goldberg, 2012) • Is a master regulator of meiotic genes related to multiple processes in spermatocytes that required for cell cycle progression (Bolcun-Filas et al., 2011)
Rad51ap1	• Its disruption blocks alternative lengthening of telomeres (ALT) activity and leads to extensive telomere shortening in ALT+cancer cell lines (Barroso-González et al., 2019) • RAD51AP1 silencing suppresses the epithelial-mesenchymal transition (EMT) and metastasis of non-small cell lung cancer (NSCLC) (Wu et al., 2019)
Pycr1	• PYCR1 is localized in the mitochondria, related to conversion of glutamate to proline (De Ingeniis et al., 2012) • A key enzyme in proline production and high levels of PYCR1 is involved in a compensatory mechanism allowing tumor expansion (Loayza-Puch et al., 2016)
Kifc1	• A highly expressed in a variety of neoplasm and promotes EMT and metastasis of hepatocellular carcinoma via gankyrin/AKT signaling (Han et al., 2019) • Involves in regulating DNA synthesis in S phase and chromatin maintenance in mitosis, and maintains cell growth in a nuclear transport-independent way (Wei and Yang, 2019)
Fzd3	• A member of the frizzled gene family and is a susceptibility locus for schizophrenia • Expresses in the mouse anterior neural tube and controls proper midbrain morphogenesis (Stuebner et al., 2010)
F13a1	• Encodes the coagulation factor XIII A subunit which is a proenzyme of plasma transglutaminase consisting of catalytic A (FXIII-A) and non-catalytic B subunits (FXIII-B) • Congenital or acquired FXIII-B deficiency may result in increased bleeding tendency through impaired fibrin stabilization (Souri et al., 2015)
Timp1	• A natural inhibitor of the matrix metalloproteinases (MMPs), a group of peptidases involved in degradation of the extracellular matrix, but able to exert multiple effects on cell growth, proliferation, differentiation and apoptosis, in an MMP-independent manner • TIMP1 deficiency exacerbates carbon tetrachloride-induced liver injury and fibrosis in mice (Wang et al., 2011) • Displays complete pro-tumor activities through its upregulation in liver tissue and serum from patients and mouse model with liver disease (Yoshiji et al., 2000) • Shows MMP-independent role of TIMP1 at the blood brain barrier during viral encephalomyelitis (Savarin et al., 2013), or regulation of adipogenesis of adipose-derived stem cells via Wnt singnaling pathway (Wang et al., 2020)
Shc4	• Involves in coupling receptor tyrosine kinases to the Ras-mitogen activated protein kinase signaling pathway, and to have a predominant cytoplasmic distribution (Ahmed and Prigent, 2014) • Acts non-canonically to promote phosphorylation of select epidermal growth factor receptor (EGFR) residues (Wills et al., 2014)
Abcc12	• Is a member of the superfamily of ATP-binding cassette (ABC) transporters and the MRP subfamily which is involved in multi-drug resistance • In all the breast cancer tissues, the expression of ABCC12 gene 3.74 times higher than in controls (Esmaeili et al., 2018)
Tubb2b	• Microtubules, which is a key participant in processes such as mitosis and intracellular transport, are composed of heterodimers of alpha- and beta-tubulins • Among taxane-based chemotherapy group, cases with higher β-tubulinIII expression were associated with a significantly more favorable prognosis compared with those having lower β-tubulinIII expression (Aoki et al., 2009)

### Validation of Five DE Genes

**Figure 1:**
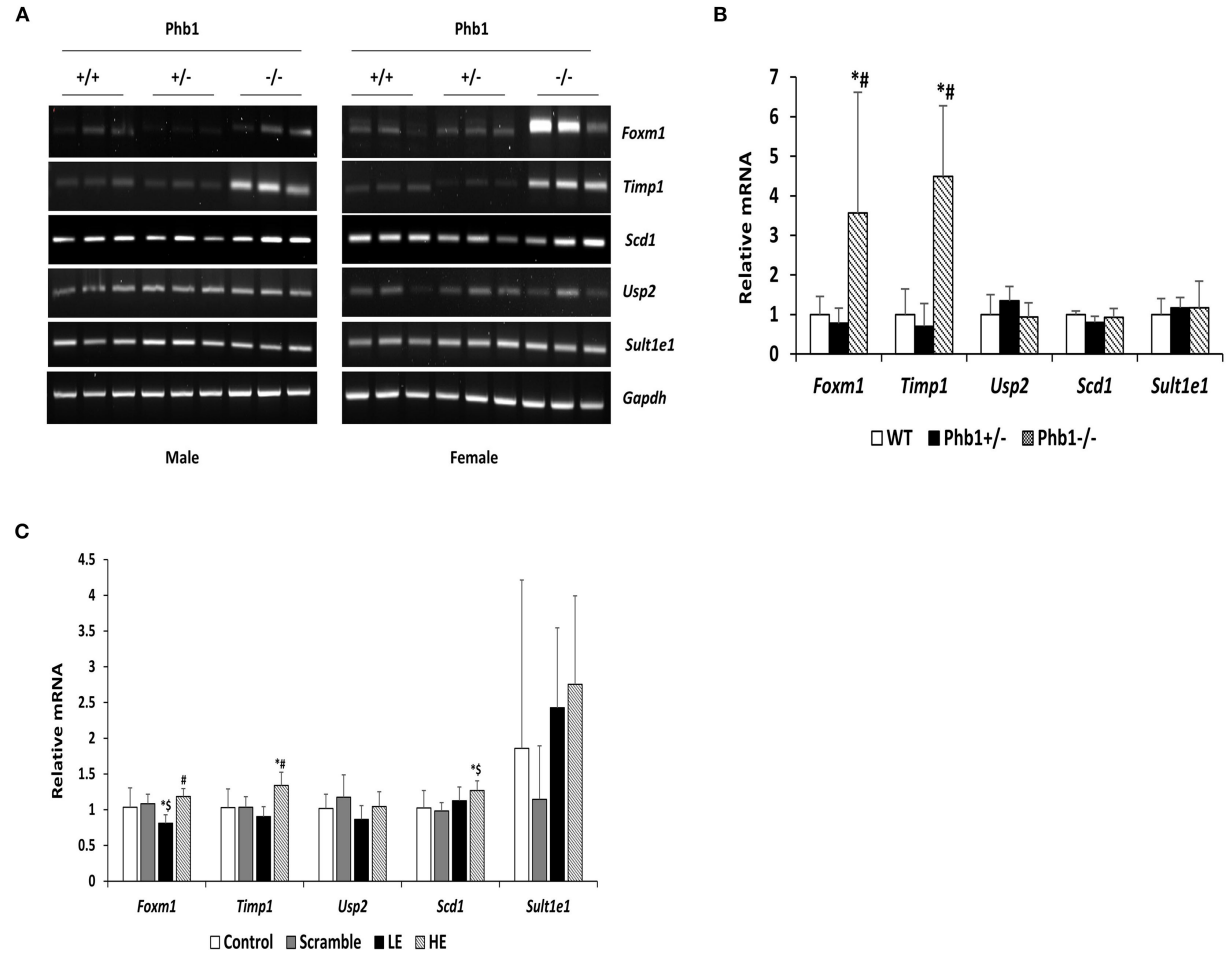
The mRNA expression levels of selected five DE genes in liver tissues and AML12 cells transfected with *Phb1* siRNA. **(A)** The mRNA expression of selected five DE genes in each group of three males and three females (*n* = 6/ genotype) was detected by RT-PCR. **(B)** Band density from RT-PCR was quantified with Image J software and represented as the fold change of control and normalized *Gapdh*. **(C)** The mRNA expression levels of selected five DE genes in AML12 cells transfected *siPhb1* (*n* = 9) were detected by qRT-PCR and normalized *b-actin*. “LE” means “low efficiency”, represented Phb1^+/−^, and “HE” means “high efficiency”, represented Phb1^−/−^. All data were expressed as means ± standard deviation. One-way ANOVA followed by Duncan's *post hoc* test was performed, and differences were considered statistically significant. *p < 0.05 vs. wild type (WT) and control, respectively. ^#^*p* < 0.05 vs. Phb1^+/−^ and low efficiency (LE). ^$^*p* < 0.05 vs. scramble.

Of 78 DE genes in the liver-specific Phb1^+/−^ group, forkhead box M1 (Foxm1), Timp1, Usp2, stearoyl-CoA-desaturase 1 (Scd1), and Sult1e1 are known to be associated with hepatic system disease (Haimerl et al., 2009; Wang et al., 2011; Hu et al., 2014; Lounis et al., 2016; Matsushita et al., 2017). We verified the five DE genes obtained RNA-seq data, using RT-PCR in the same liver tissues, in addition to qRT-PCR in normal murine hepatocytes transfected with si*Phb1*. Phb1 was silenced by si*RNAs*, which mimic Phb1^−/−^ and Phb1^+/−^ (Supplementary Materials 4, 5). The mRNA expression levels of all 5 DE genes were well-matched between Phb1^+/−^ and WT in terms of the direction (Table 3; Figures 1A,B). But, in *in vitro* validation, the mRNA expression levels of *Foxm1* and *Timp1* were only matched with RNA-seq and RT-PCR validation in liver tissues (Table 3; Figure 1C), which may be due to differences between animal tissues and cell culture. These results confirmed both validity of RNA-seq data for further pathway analyses and the reliability of our liver disease model, indicating that half deficiency of Phb1 in the liver can elucidate the altered molecular mechanisms related to liver injuries.

**Table 3:** The selected 5 DE genes in comparison between Phb1^−/−^, Phb1^+/−^, and WT.

**Gene symbol**	**Hetero vs. WT**	**KO vs. WT**	**KO vs. Hetero**
	RNA-seq^*^		
Foxm1^**^	**−3.1**	**7.9**	**24.3**
Timp1^**^	**−20.6**	**86.1**	**1,769.3**
Usp2	**2.4**	2.2	**−3.6**
Scd1	**−2.6**	**−2.8**	−1.1
Sult1e1	**6.6**	**5.4**	−1.2

“*Hetero” and “KO” in Tables represent “Phb1^+/−^” and “Phb1^−/−^”, respectively*.

* *Values mean linear fold changes and only statistically significant values (FDR <0.05) are in bold*.

** *Consistent with validation for RNA-seq by RT-PCR from liver tissues and qPCR from AML12 cells transfected with Phb1 siRNA*.

### Bioinformatic Pathway Analyses: Upstream Regulators and Molecular Networks

**Figure 2:**
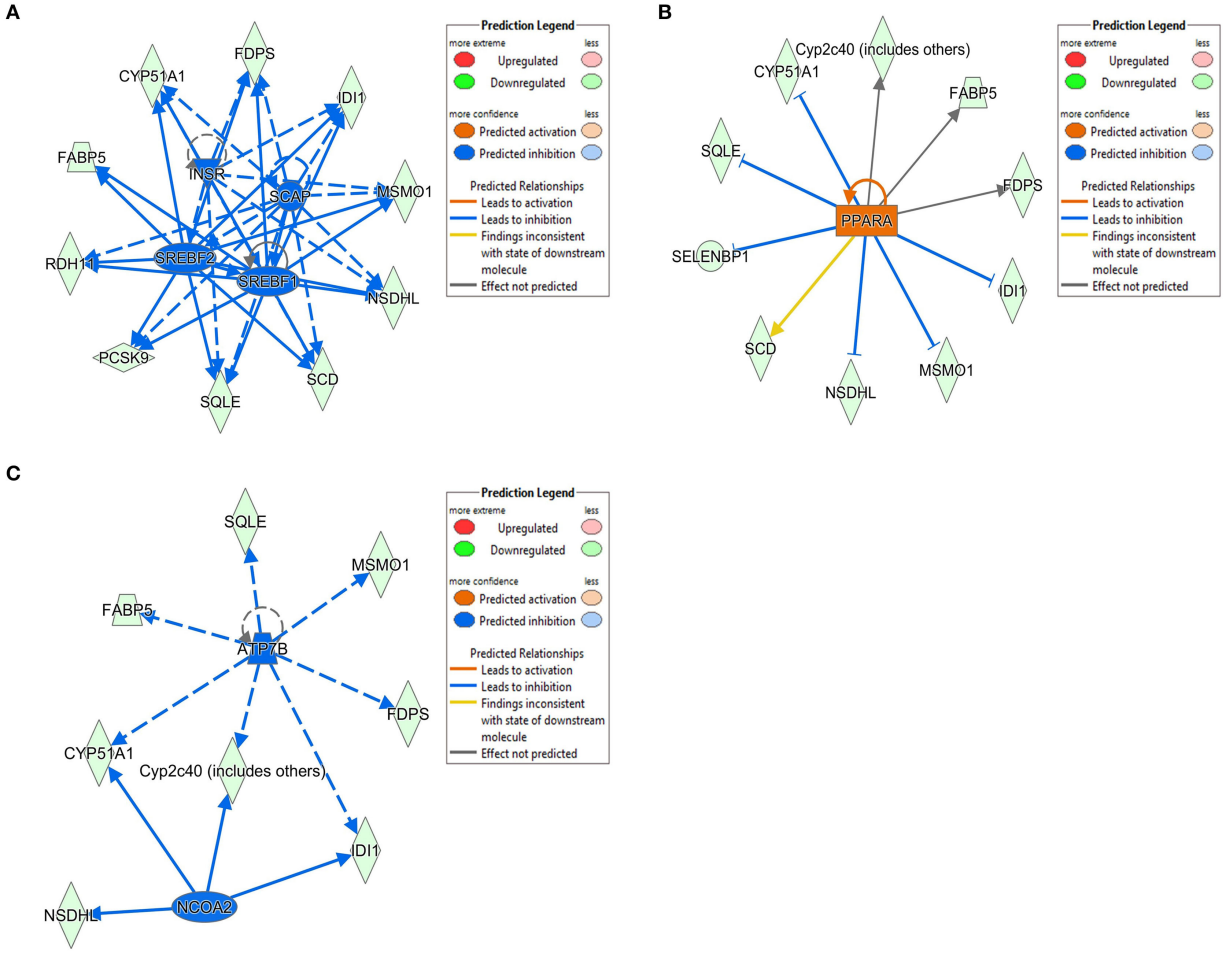
DE genes between Phb1^+/−^ and WT, regulated by **(A)** Insr, Scap, Srebf1, and Srebf2; **(B)** Pparα; **(C)** Atp7b and Ncoa2. Red, green, orange, and blue represent upregulation, downregulation, predicted upregulation, and predicted downregulation, respectively.

**Figure 3:**
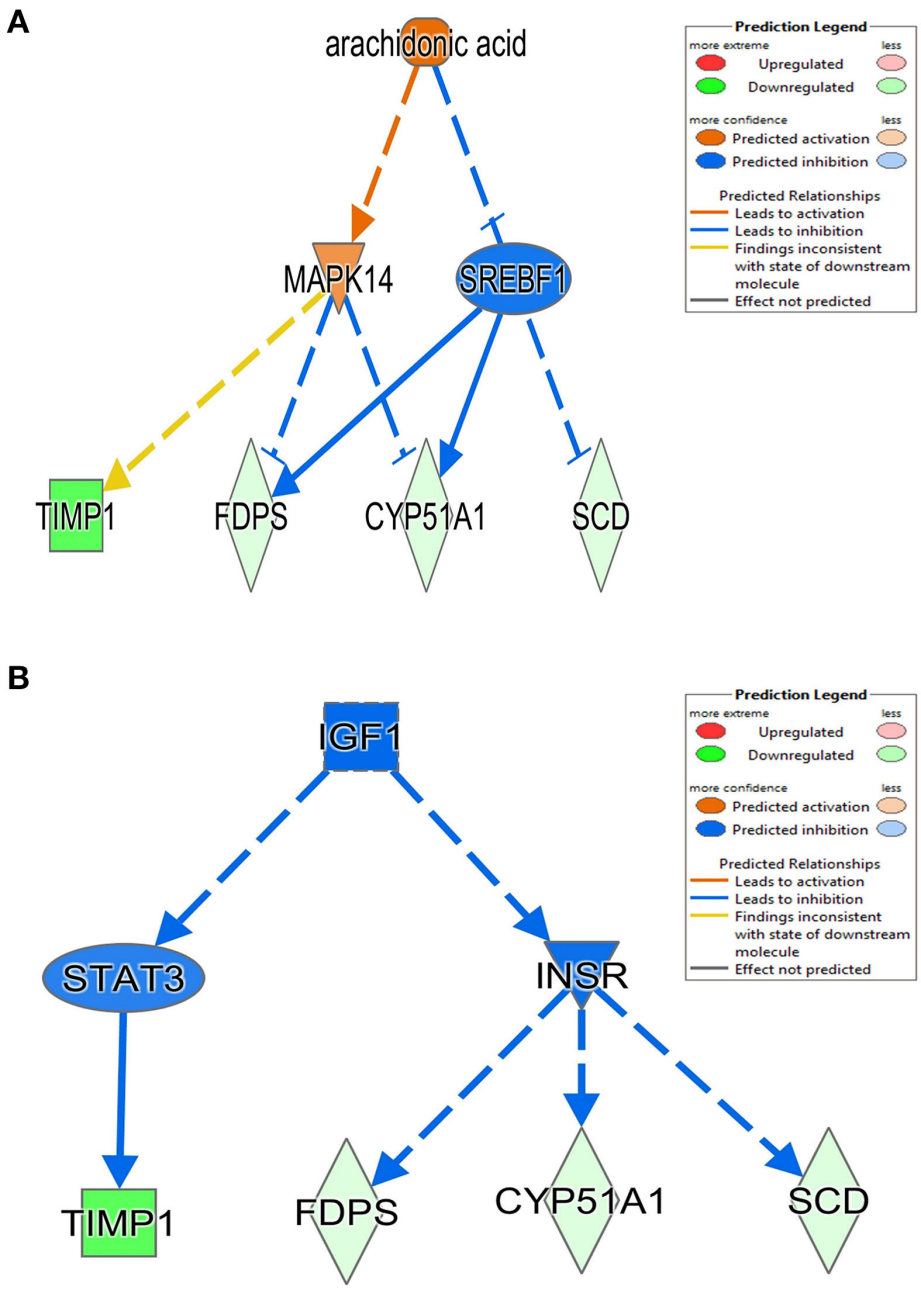
Causal network between Phb1^+/−^ and WT, regulated by **(A)** arachidonic acid and **(B)** Igf1. Red, green, orange, and blue represent upregulation, downregulation, predicted upregulation, and predicted downregulation, respectively.

The 78 DE genes in Phb1^+/−^ compared with WT were subjected to core pathway analyses, using ingenuity pathway analysis (IPA) software. Upstream regulator analysis was conducted with enriched pathways and *p*-value computed by IPA literature review algorithm. The resulting causal networks were scored for known physiological relationships with diseases, functions, genes, or chemicals in the liver tissue and liver cell lines. Causal networks derived by participating the regulator molecule, which controls the expression effector molecules in the given dataset. Thus, causal networks are small hierarchical networks of regulators that control the expression of the given data set. Upstream regulator analyses containing networks provide positive and negative *z*-scores (≥2 and ≤−2, respectively), which are considered significant predictions of activation and inhibition, respectively. Results showed that sterol regulatory element-binding transcription factor 2 (Srebf2) (*z*-score = −3.12), Srebf1 (*z*-score = −3.08), SREBP cleavage-activating protein (Scap) (*z*-score = −2.98), insulin receptor (Insr) (*z*-score = −2.62), ATPase copper transporting beta (Atp7b) (*z*-score = −2.65), and nuclear receptor coactivator 2 (Ncoa2) (*z*-score = −2.00) were predicted to be inhibited, while peroxisome proliferator-activated receptor alpha (Pparα) (*z*-score = 2.16) was predicted to be activated in liver of Phb1^+/−^ mice (Table 4). Figure 2 showed these three potential interactions were centered with: (1) Scap, Srebf1, Srebf2; (2) Atp7b, Ncoa2; (3) Pparα, Ncoa2. Similarly, causal network regulator analysis showed that Srebf2 (*z*-score = −3.16), Srebf1 (*z*-score = −3.16), Scap (*z*-score = −3), Insr (*z*-score = −2.65), Atp7b (*z*-score = −2.65), insulin-like growth factor 1 (Igf1) (*z*-score = −2.53) were predicted to be inhibited, while arachidonic acid (*z*-score = 2.31) was predicted to be activated (Table 5). Srebf1, Srebf2, and Scap, which are involved in regulating cellular lipid metabolism, were listed as the highest ranks of causal network regulators. The predicted downstream target molecules of arachidonic acid were mitogen-activated protein kinase 14 (Mapk14) and Srebf1, followed by downregulating molecules involved in cholesterol and triglycerides (TG) synthesis such as farnesyl diphosphate synthase (Fdps), cytochrome P450 family 51 subfamily A member 1 (Cyp51a1), and Scd1 (Figure 3A). Igf1 was predicted to be inhibited and downregulated similar genes associated with cholesterol and triglycerides *via* Insr (Figure 3B). Also, Timp1 is one of the target molecules of Igf1 *via* signal transducer and activator of transcription 3 (Stat3) (Figure 3B).

**Table 4:** Upstream regulators between Phb1^+/−^ and WT.

**Upstream regulator**	**Activation *z*-score**	***p*-value of overlap**	**Target molecules in dataset**
SREBF2	−3.12	5.42E-16	CYP51A1, FABP5, FDPS, IDI1, MSMO1, NSDHL, PCSK9, RDH11, SCD, SQLE
SCAP	−2.98	3.49E-14	CYP51A1, FDPS, IDI1, MSMO1, NSDHL, PCSK9, RDH11, SCD, SQLE
INSR	−2.62	7.27E-13	CYP51A1, FDPS, IDI1, MSMO1, NSDHL, SCD, SQLE
SREBF1	−3.08	1.04E-12	CYP51A1, FABP5, FDPS, IDI1, MSMO1, NSDHL, PCSK9, RDH11, SCD, SQLE
ATP7B	−2.65	1.4E-11	Cyp2c40 (includes others), CYP51A1, FABP5, FDPS, IDI1, MSMO1, SQLE
PPARA	2.16	3.37E-08	Cyp2c40 (includes others), CYP51A1, FABP5, FDPS, IDI1, MSMO1, NSDHL, SCD, SELENBP1, SQLE
POR		4.88E-08	CYP51A1, FDPS, IDI1, MSMO1, NSDHL, SCD, SQLE
MAPK14	0.15	1.05E-06	COL1A1, CYP51A1, FDPS, TIMP1
Ethanol	−0.71	1.12E-05	ACTA2, NSDHL, PCSK9, SCD, SQLE, TIMP1
NCOA2	−2	1.13E-05	Cyp2c40 (includes others), CYP51A1, IDI1, NSDHL

**Table 5:** Causal network regulators between Phb1^+/−^ and WT.

**Master regulator**	**Molecule type**	**Activation *z*-score**	***p*-value of overlap**	**Target molecules in dataset**
SREBF2	Transcription regulator	−3.16	2.07E-16	CYP51A1, FABP5, FDPS, IDI1, MSMO1, NSDHL, PCSK9, RDH11, SCD, SQLE
Arachidonic acid	Chemical - endogenous mammalian	2.31	2.16E-16	COL1A1, CYP51A1, FABP5, FDPS, IDI1, MSMO1, NSDHL, PCSK9, RDH11, SCD, SQLE, TIMP1
NR1H4	Ligand-dependent nuclear receptor	−1.73	9.98E-15	ACTA2, CYP51A1, FABP5, FDPS, FOXM1, IDI1, MSMO1, NSDHL, SCD, SQLE, SULT1E1, TIMP1
IGF1	Growth factor	−2.53	2.69E-14	ACTA2, COL1A1, CYP51A1, FDPS, IDI1, MSMO1, NSDHL, SCD, SQLE, TIMP1
SREBF1	Transcription regulator	−3.16	2.69E-14	CYP51A1, FABP5, FDPS, IDI1, MSMO1, NSDHL, PCSK9, RDH11, SCD, SQLE
SCAP	Other	−3	2.81E-14	CYP51A1, FDPS, IDI1, MSMO1, NSDHL, PCSK9, RDH11, SCD, SQLE
BTG1	Transcription regulator	−1.67	6.46E-14	ACTA2, COL1A1, CYP51A1, FDPS, IDI1, MSMO1, NSDHL, SCD, SQLE
PTPN1	Phosphatase	1.67	5.3E-13	COL1A1, CYP51A1, FDPS, IDI1, MSMO1, NSDHL, SCD, SQLE, TIMP1
INSR	Kinase	−2.65	7.27E-13	CYP51A1, FDPS, IDI1, MSMO1, NSDHL, SCD, SQLE
ATP7B	Transporter	−2.65	1.4E-11	Cyp2c40 (includes others), CYP51A1, FABP5, FDPS, IDI1, MSMO1, SQLE

**Figure 4:**
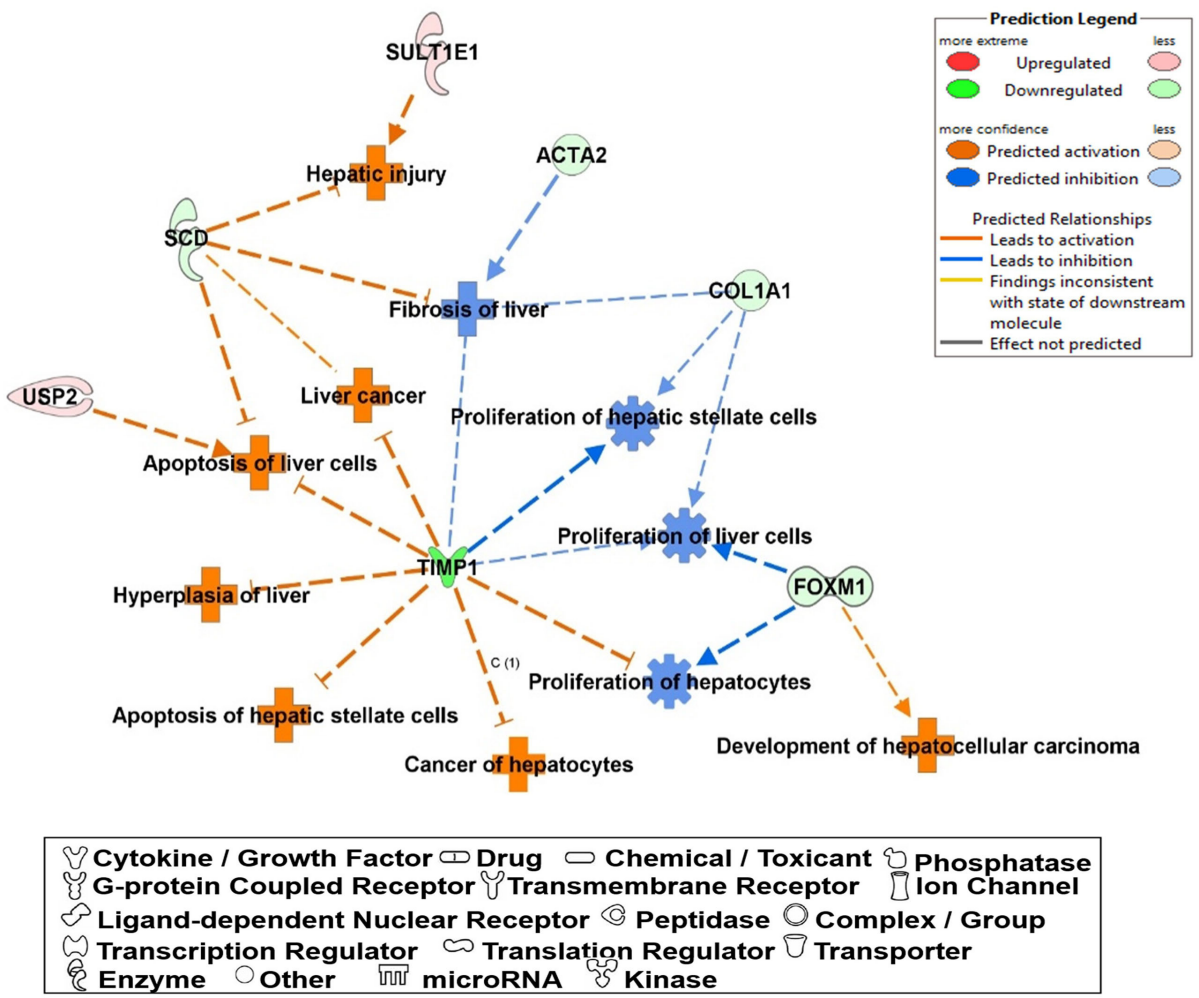
A network of DE genes affecting functions related to “hepatotoxicity” between Phb1^+/−^ and WT. Red, green, orange, and blue represent upregulation and downregulation, predicted upregulation, and predicted downregulation, respectively.

The most critical diseases and biofunctions (Table 6), molecular and cellular functions (Table 7), and physiological system development and functions (Table 8) were identified with influences of 78 DE genes, and specific genes involved in all diseases and functions were listed in Supplementary Material 6. Of diseases and biofunctions listed in Table 6, hepatic system diseases were ranked as the second. The molecules, including Scd1, Acta2, collagen type I alpha 1 chain (Col1a1), Timp1, Foxm1, Sult1e1, and plasminogen activator (Plat), are also involved in hepatotoxicity pathways, such as liver fibrosis, liver hyperplasia/hyperproliferation, liver proliferation, and liver necrosis/cell death (Table 9). Molecules associated with hepatotoxicity were shown in Figure 4. Hepatic DE genes in Phb1^+/−^ mice showed increased risks of hyperplasia of the liver, apoptosis of liver cells, hepatic injury, and hepatoma and decreased functions in the proliferation of liver cells and fibrosis. The hepatotoxicity analysis indicates that Phb1^+/−^ mice may become more susceptible with liver damages/injuries.

**Table 6:** Top diseases and biofunctions between Phb1^+/−^ and WT.

**Name**	***p*-value range**	**Molecules**
Gastrointestinal disease	4.87E-02 – 2.46E-04	ACTA2, COL1A1, SCD, TIMP1, FOMX1, PBK, PLAT, USP2
Hepatic system disease	4.87E-02 – 2.46E-04	ACTA2, COL1A1, SCD, TIMP1, FOXM1, USP2, PLAT
Organismal injury and abnormalities	4.87E-02 – 2.46E-04	ACTA2, COL1A1, SCD, TIMP1, PBK, PLAT, FOXM1, USP2
Cancer	1.77E-02 – 1.21E-03	ACTA2, PBK, PLAT, TIMP1, FOXM1
Cardiovascular disease	4.87E-02 – 3.56E-03	FOXM1, PLAT

**Table 7:** Molecular and cellular functions between Phb1^+/−^ and WT.

**Name**	***p*-value range**	**Molecules**
Carbohydrate metabolism	4.19E-02 – 3.56E-03	SCD
Cell-to-cell signaling and interaction	3.56E-03 – 3.56E-03	SCD
Cellular assembly and organization	3.56E-03 – 3.56E-03	SCD
Lipid metabolism	4.87E-02 – 3.56E-03	SCD, FABP5
Molecular transport	4.97E-02 – 3.56E-03	SCD, FABP5

**Table 8:** Physiological system development and function between Phb1^+/−^ and WT.

**Name**	***p*-value range**	**Molecules**
Cardiovascular system development and function	1.40E-02 – 1.74E-03	COL1A1, FOXM1, TIMP1
Digestive system development and function	4.53E-02 – 3.56E-03	FOXM1, COLA1A1, TIMP1, SCD, PLAT
Hepatic system development and function	4.53E-02 – 3.56E-03	FOXM1, COLA1A1, TIMP1, SCD, PLAT
Organ morphology	4.53E-02 – 3.56E-03	FOXM1, PLAT
Organismal development	4.53E-02 – 3.56E-03	FOXM1, COL1A1, TIMP1

**Table 9:** Hepatotoxicity between Phb1^+/−^ and WT.

**Name**	***p*-value range**	**Molecules**
Liver fibrosis	1.36E-01 – 2.46E-04	SCD, ACTA2, COL1A1, TIMP1
Liver hyperplasia/hyperproliferation	1.00E00 – 3.56E-03	TIMP1
Liver proliferation	1.06E-01 – 1.40E-02	FOXM1, COL1A1, TIMP1
Liver necrosis/cell death	8.80E-02 – 2.12E-02	TIMP1
Liver damage	6.94E-02 – 6.94E-02	SCD, SULT1E1

**Figure 5:**
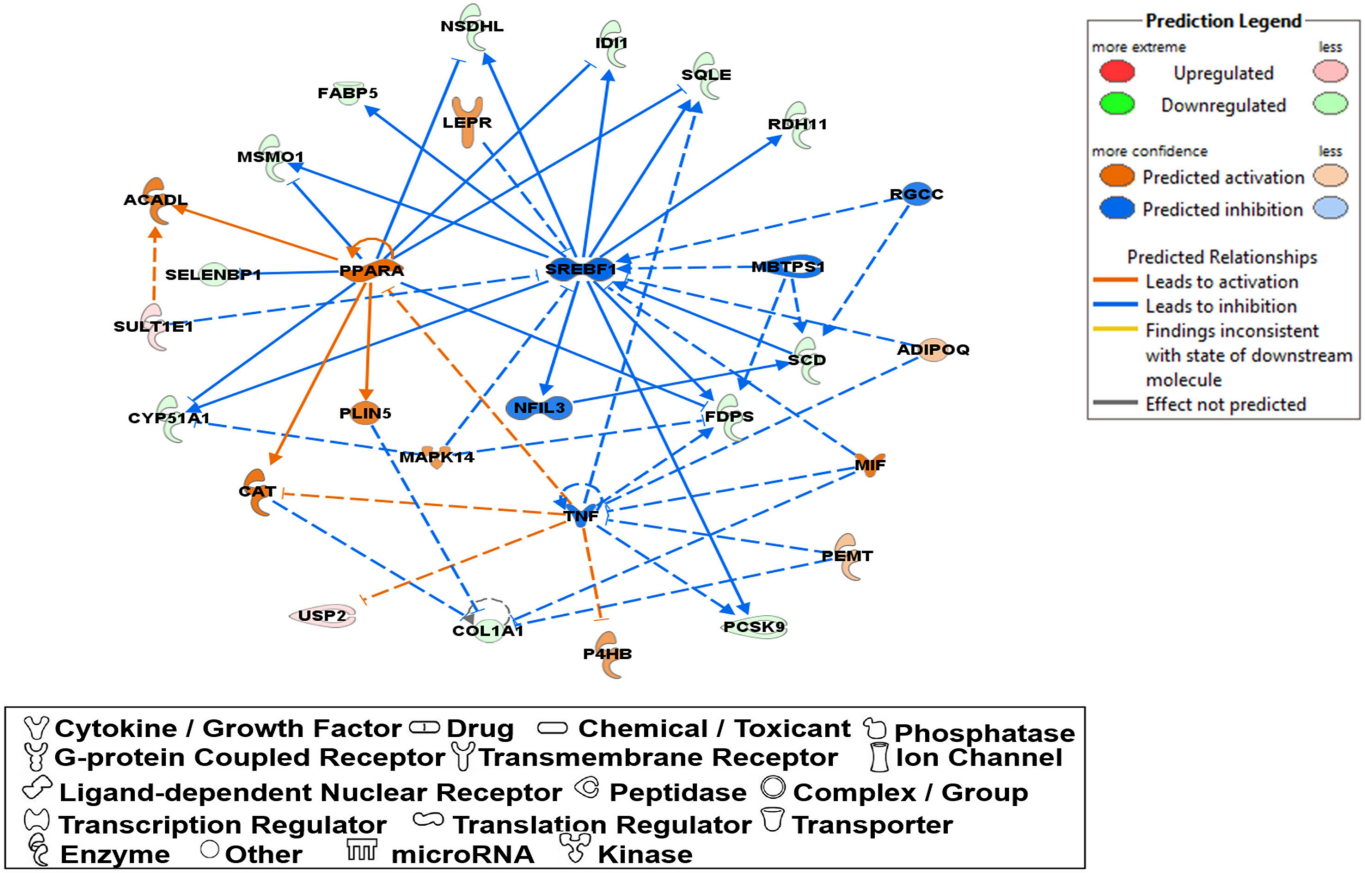
A network relevant to “lipid metabolism”, “small molecule biochemistry”, “vitamin and mineral represented in a gene network (network ID 1). Red, green, orange, and blue represent upregulation and downregulation, and predicted upregulation and downregulation, respectively.

Molecular network analysis produced five potential molecular interactions among 78 DE genes (Table 10). The most interesting network #1 centered with regulatory molecules, including Pparα and Srebf1, which were discussed in upstream regulators above, in addition to tumor necrosis factor (TNF), which is predicted to be inhibited. Molecules interacting network #1 are connected to functions of lipid metabolism, small molecule biochemistry, and vitamin and mineral metabolism (Table 10; Figure 5).

**Table 10:** Molecular networks between Phb1^+/−^ and WT.

**Network_ID**	**Molecules in network**	**Score**	**Focus molecules**	**Top diseases and functions**
1	ACADL, ADIPOQ, CAT, CLOCK, COL1A1, CYP51A1, Cyp2c40 (includes others), DBI, FABP5, FDPS, IDI1, IGFBP4, LEPR, MAPK14, MBTPS1, MIF, MSMO1, NFIL3, NSDHL, Ncoa6, P4HB, PCSK9, PEMT, PER2, PLIN5, PPARA, RDH11, RGCC, SCD, SELENBP1, SQLE, SREBF1, SULT1E1, TNF, USP2	28	16	Lipid metabolism, small molecule biochemistry, vitamin and mineral metabolism
2	ABCB4, ACTA2, APOE, CYP7A1, FABP1, FOXM1, HGF, HIF1A, KLF11, LEP, MIF, MMP2, NR1H4, PDGFC, PLAT, S1PR2, STAT3, TGFB1, TIMP1, ethanol	5	4	Cellular movement, hematological system development and function, immune cell trafficking
3	RASSF1, TUBB2B	2	1	Cell cycle, cellular assembly and organization, cellular function and maintenance
4	IFI16, MTOR, TNK1	2	1	Cancer, endocrine system disorders, organismal injury and abnormalities
5	GSTA1, GSTA3, Gsta1, MAF	2	1	Drug metabolism, glutathione depletion in liver, endocrine system development and function

## Discussion

In this study, we examined the hepatic transcriptome of liver-specific Phb1 deficient mice and identified biological functions and genes associated with hepatotoxicity, lipid metabolism, and metabolic disorders. IPA suggested a network related to hepatotoxicity, which is composed of Foxm1, Timp1, Usp2, Scd1, and Sult1e1. These data exhibited that decreased Phb1 in hepatocytes contributed to abnormal proliferation of various liver cell types and cancer cell transformation by upregulated or downregulated genes. Also, IPA analysis with DE genes suggested increasing arachidonic acid and suppressing Igf1-mediated signaling as causal networks in liver-specific Phb1^+/−^, compared with WT. They had in common with downstream targets as Timp1, Scd1, Fdps, and Cyp51a1 (Figure 3), which are mainly associated with the extracellular matrix, fatty acid metabolism, and steroid biosynthesis (Ge et al., 2020; Yan et al., 2020) and downregulated *via* Mapk14, Srebf1, Stat3, and Insr. This finding is in line with the blocking of activation of phosphoinositide 3-kinase by insulin *via* p38 Mapk in hepatocytes with arachidonic acid (Talukdar et al., 2005) and evidence that arachidonic acid has the potential to decrease insulin-mediated activation of Srebp-1c by inhibiting liver X receptor (Lxr) activation in rat hepatocytes (Chen et al., 2004). Also, Igf1 and Insr-mediated Cyp51a1 were predicted as a downregulated enzyme, and related research reported that hepatocyte Cyp51KO mice showed inflammation and mild-to-moderate portal fibrosis and abnormal hepatic sterol metabolism (Urlep et al., 2017). Collectively, these results may elucidate physiological changes by Phb1 and provide a comprehensive understanding of prognosis and prevention of liver injuries, containing liver cancer.

### Liver Disease-Related Genes

Of 78 DE genes, which were filtered out from liver-specific Phb1^+/−^ vs. WT, Foxm1, Timp1, Usp2, Scd1, Sult1e1, are known to be associated with hepatotoxicity (Figure 4). Foxm1, a transcription regulator, is located to the nucleus and highly expressed in proliferating normal cells and various cancer cells (Teh, 2012). The knockdown of FOXM1 in human hepatocellular carcinoma (HCC) cell lines significantly alleviated cell cycle arrest and cell growth suppression (Hu et al., 2014). In addition, overexpression of FOXM1 is associated with an aggressive tumor feature and poor prognosis of HCC (Sun et al., 2011). Unlike earlier results of positive correlation between Foxm1 and HCC, our transcriptomic profile demonstrated that fold change of Foxm1 was rather reduced in Phb1^+/−^ compared with WT (Phb1^+/−^ vs. WT, log_2_ FC = −1.6) (Supplementary Material 3) and then increased in Phb1^−/−^ compared with Phb1^+/−^ (Phb1^−/−^ vs. Phb1^+/−^, log_2_ FC = 4.6) (data not shown). This finding was consistent with validation using the PCR method in liver tissues and AML12 cells transfected with si*Phb1* (Figure 1). Deletion of Foxm1 is correlated with Ras-induced HCC with stem cell features by accumulating reactive oxygen species (ROS) (Kopanja et al., 2015). Another study revealed a negative association with Foxm1/nuclear factor kappa B (NF-kB) and methionine adenosyltransferase 1A (Mat1a) that is responsible for catalyzes in the conversion of methionine to homocysteine (Li et al., 2020). It was reported that Foxm1 directly interacts with Mat1a, which is a tumor suppressor in the liver. Collectively, it can be interpreted that Phb1 leads to a unique change of the expression pattern of Foxm1 and may be a key regulator, modulating the transcriptional activity of Foxm1 in terms of tumorigenesis.

Likewise, Timp1 expression in the transcriptomic profile and PCR validation was similar to the results of Foxm1 expression. Previous studies explained increasing Timp1 expression as a marker of extrahepatic and intrahepatic tumors from liver tissues and serum (Ylisirniö et al., 2000; Yoshiji et al., 2000; Yukawa et al., 2007). Also, Thiele et al. (2017) suggested that hepatic Timp1 mRNA expression from WT mice was upregulated in HCC tissue compared with adjacent paired normal tissue when co-treated with diethylnitrosamine and CCL_4._ In agreement with these studies, our transcriptomic profile showed increased Timp1 expression in Phb1^−/−^, compared with Phb1^+/−^ (log_2_ FC = 10.8) (Table 1). On the contrary, Timp1 expression was rather decreased in Phb1^+/−^, compared with WT, which was similar to Foxm1 (log_2_ FC = −4.4) (Table 1). Wang et al. (2011) demonstrated the newly hepatoprotective role of Timp1 during acute and chronic liver injuries, which is positively regulated by the interleukin-6 (IL-6)/STAT3 signaling pathway. Our liver-specific Phb1-deficient mouse showed different Timp1 expression patterns according to Phb1 depletion, which encompassed both protumor- and antitumor activity of Timp1. It was previously reported that Timp1 was increased in the liver from adipocyte-specific nuclear form of SREBP-1c (nSREBP-1c) transgenic mice, showing human nonalcoholic steatohepatitis (NASH) (Kakino et al., 2018). However, Upregulation of Timp1 was reduced in the liver from the Tnf^−/−^ nSREBP-1c transgenic mice (Kakino et al., 2018). Tomita et al. explained that control mice-fed-MCD diet rapidly increased mRNA expression of Timp1 in the whole liver, compared with both TNF receptors 1 (Tnfr1) and 2 (Tnfr2) knockout mice, representing Tnfr-double KO mice (Tomita et al., 2006). We can interpret that decreased Timp1 may act as an initial signal of liver injuries by Phb1^+/−^, and then chronic liver injuries by Phb1^−/−^ may represent a rapid increase of Timp1. Since we recognize the importance to uncover a direct correlation between Phb1 and Timp1, further study is underway to assess the association. Nevertheless, both RNA-seq and PCR validation in siPhb1-transfected hepatocytes showed indirect inverse relevance between Phb1 and Timp1 in this research. Especially, Ko et al. already explained the relation using microarray analyses (Ko et al., 2010), showing an increase in the expression of Timp1 in liver-specific Phb1^−/−^ mice. Therefore, Timp1 may be an important enzyme that predicts the extent of liver injuries by lack of the Phb1 gene. Further investigations are needed to characterize functional connections between Phb1 and Timp1 in the liver disease model. Despite increased expression of Foxm1 and Timp1 in an environment with liver injuries, another possibility to interpret the differential gene expression in Phb1^+/−^ vs. Phb1^−/−^ may be deciphered by genetic compensation by the gene knockout (El-Brolosy and Stainier, 2017). Genetic compensation means that another gene takes over the role of the knocked-out gene (De Souza et al., 2006). This concept is used for explaining the discrepancy between knockdown and knockout phenotypes. For example, it was reported that upregulated Emilin 3, which shares the functional domain with egfl7 represented for endothelial extracellular matrix genes, was observed only in the zebrafish egfl7 knockout, not in the knockdown, demonstrating that Emilin 3 may compensate for the egfl7 knockout (Rossi et al., 2015). Besides, both Prnp and Sprn proteins share biological functions in early embryogenesis according to the need for either Prnp or Sprn expression, showing that one can compensate for the absence of the other in PrP-knockout mammals (Young et al., 2009). According to these cases in which gene expression is reversely regulated in the knockout and knockdown comparison of the unique expression pattern of Foxm1 and Timp1 in Phb1^+/−^ and Phb1^−/−^ may be explained by genetic compensation. To specify the concept in our liver disease model, further intensive research is necessary in the environment with regulating the expression of Phb1.

Ubiquitin carboxyl-terminal hydrolase 2 (Usp2), which is upregulated in Phb1^+/−^ compared with WT (log_2_ FC = 1.3) (Table 1), is a deubiquitinating enzyme and involved in controlling activity and levels of protein under certain physiological conditions. Usp2 has been reported to stabilize mouse double minute 2 (Mdm2) and Mdm4, which are proto-oncogenes with both p53-dependent or independent activities, to degrade p53 (Benassi et al., 2012; Young et al., 2019), maintaining the expression of p53 at a low level. Specifically, protein-protein interaction assays using the bacterial two-hybrid system showed that USP2 can deubiquitinate MDM2 and promotes p53 degradation, showing the association between USP2 and MDM2 (Stevenson et al., 2007). Especially, it was reported that transfection of tumor-derived cell lines with si*Usp2* resulted in increasing p53 protein expression and its target gene, p21 (Stevenson et al., 2007). PHB1 has an ability to physically interact with p53 and enhance p53-mediated transcriptional activation by promoting its recruitment to promoters in two breast cancer cell lines where it co-localizes with p53 (Fusaro et al., 2003). Our transcriptome analysis showed that the expression level of Usp2 was upregulated in Phb1^+/−^ compared with WT (log_2_ FC = 1.3) and then decreased in Phb1^−/−^ compared with Phb1^+/−^ (log_2_ FC = −1.8) (Table 1). We can infer that Phb1 may participate in enhancing Usp2-mediated proteosome activity, and Phb1 deficiency may result in abnormal p53 stabilization and activation (Haupt et al., 1997; Honda and Yasuda, 2000). Thus, we can explain that there is no defense response against a high-stress environment, such as an increasing susceptibility of liver injuries by the drastic shortage of Phb1, represented to Phb1^−/−^.

Stearoyl-CoA desaturase-1 (SCD1) plays an important role in the conversion of saturated fatty acids to monosaturated fatty acids and further synthesis of TG (Lounis et al., 2016). We expected that Scd1 expression in Phb1^+/−^, compared with WT, also increased in transcriptome analysis, since our previous research on the effect of Phb1 deficiency with si*Phb1* knockdown on impaired lipid metabolism demonstrated that palmitic acid promoted *Scd1* mRNA expression levels (unpublished data, under review). In the current study, Scd1 expression was decreased in Phb1^+/−^ compared with WT (log_2_ FC = −1.4) (Supplementary Material 3), and there was no statistically significant difference between Phb1^+/−^ and Phb1^−/−^ (Table 3). Wang et al. (2002a) reported that PHB1-mediated transcriptional repression required histone deacetylase (HDAC), and additional corepressors like N-CoR are involved. Also, E2F transcription factors, which play a key role in regulating mammalian cell cycle progression, were controlled by PHB1, interacting with retinoblastoma protein (Rb) (Wang et al., 2002b; Mishra et al., 2006). Recently, combining research using lipidomics and transcriptome analyses in Rb depletion in mouse embryonic fibroblast has shown that Rb deficiency increases the concentration of fatty acid, acylcarnitine, phosphatidylcholine, and arachidonoyl ethanolamine (Muranaka et al., 2017). Also, Scd1 is most strongly controlled by Rb, possibly through E2F and SREBP transcription factors (Muranaka et al., 2017). Collectively, a decrease in Scd1 expression in Phb1^+/−^ compared with WT may be due to the interaction between PHB1 and SCD1 at the protein level, maintaining a balance between lipid catabolism and anabolism.

Sulfotransferase family 1E member 1 (SULT1E1) is a cytosolic enzyme, which is called as an estrogen sulfotransferase and is responsible for the inactivation of β-estradiol (E2) involved in changing estrogen metabolism and liver function (Li and Falany, 2007; Li et al., 2009; Xu et al., 2018). According to a study on cystic fibrosis, which is an inherited disorder and affects the lung, intestine, pancreas, and livers, hepatic Sult1e1 activity was increased in cystic fibrosis transmembrane conductance regulator (CFTR) ^−/−^ mice compared with CFTR^+/+^ (Li and Falany, 2007). Also, the estrogen receptor α protein level was reduced in CFTR^−/−^mice with high Sult1e1 activity, showing high affinity between E2 and substrate of SULT1E1 and thereby altering the level of estrogen-regulated proteins (Song, 2001; Li and Falany, 2007). Wang et al. (2004) demonstrated the association of PHB1 with E2F transcription factor 1 (E2F1) and the repressive function of estrogen antagonists in human breast cancer cells (Wang et al., 2004). Moreover, the PHB1 gene and protein expression were both markedly enhanced by estrogen antagonists (Wang et al., 2004). In agreement with these studies, Sult1e1 expression in the current transcriptomic profile was increased by depletion of Phb1 in between Phb1^+/−^ and Phb1^−/−^ compared with WT (log_2_ FC = 2.7 and 2.4, respectively) (Supplementary Material 3). Both transcriptome analyses and validation, using AML12 cells transfected with si*Phb1*, showed a negative relationship between Phb1 and Sult1e1 (Figure 1C). We can infer that Phb1 deficiency is likely to have relevance to increasing expression of Sult1e1. Also, Phb1 depletion may be a crucial status to elevate hepatic Sult1e1 activity responsible for repressing estrogen-related genes in terms of its transcriptional activities. Thus, the association of Phb1 depletion with its effect on estrogen metabolism in livers needs to be investigated further.

### Lipid Metabolism and Metabolic Diseases

In addition to the hepatotoxicity of 5 DE genes between liver-specific Phb1^+/−^ and WT, there are some associations with lipotoxicity. The role of fatty acid metabolism in cancer initiation, progression, and drug resistance through the FOXO3-FOXM1 axis had been mentioned (Saavedra-García et al., 2018). Besides, compared with adipose tissue from controls, toll-like receptor 2 (Tlr2) KO mice fed a high-fat diet that decreased levels of Timp1, collagen 1, and transforming growth factor-β1 (TGFβ1) (Song et al., 2018). Usp2 enhances the stability of fatty acid synthase (FASN) by impeding proteasome-dependent degradation in human HCC (Calvisi et al., 2011; Kitamura and Hashimoto, 2021). The high-fat diet-induced NAFLD rat model showed increased protein levels of SULT1E1, compared with mice-fed normal standard diet through proteomics analysis and western blots analysis (Cong et al., 2021). From upstream regulator analysis and causal network regulators, Insr, Scap, Srebf1, and Srebf2 were predicted to be inhibited in liver-specific Phb1^+/−^ compared with WT (Tables 4, 5). SREBPs regulate the biosynthesis of triglyceride (TG), fatty acids (FAs), and cholesterol, which can increase the expression of genes related to lipid synthesis and lipid uptake (Brown and Goldstein, 1997). SREBF1 and SREBF2 encode three SREBP isoforms: SREBP-1a, SREBP-1c, and SREBP-2, respectively. SREBP-1a and−1c are usually expressed in the liver, adipose tissue, and adrenal gland of mice and humans, whereas SREBP-2 is ubiquitously expressed in cell lines, spleen, and intestinal tissues (Jeon and Osborne, 2012; Moslehi and Hamidi-Zad, 2018). When cellular sterol levels are high, SCAP, which is called ER membrane protein, interacts with SREBPs in the endoplasmic reticulum (ER) membrane, and the SREBP/SCAP complex moves to Golgi to translocate to the nucleus and bind to the target gene promoters (Xiao and Song, 2013; Moslehi and Hamidi-Zad, 2018). SREBP-1a and−1c mostly activate fatty acid and TG synthesis, and SREPB-2 increases transcription of genes related to cholesterol synthesis and uptake. Remarkably, SREBP-1c is known to be controlled by insulin treatment. Hegarty et al. (2005) showed that full induction of mature and transcriptionally active form of SREBP-1 in rat hepatocyte resulted from insulin treatment (Hegarty et al., 2005). Many studies reported that insulin is one of the most potent activators of SREBP-1c (Shimomura et al., 1999; Azzout-Marniche et al., 2000; Zeng et al., 2017). We found that Insr, SREBPs, and Scap were predicted to be inhibited in liver-specific Phb1^+/−^, compared with WT (Tables 4, 5). Potential decrease of Insr may interpret Insr-related insulin resistance in non-adipose tissues like the liver, contributing to metabolic diseases. The liver regulates various metabolism by insulin, which is triggered by INSR (Samuel and Shulman, 2012; Knebel et al., 2018). Liver-specific Insr-KO mice showed dramatic insulin resistance, abnormal glucose homeostasis, and hyperinsulinemia (Michael et al., 2000; Miao et al., 2014). In addition, according to a study on patients with simple steatosis and NASH, downregulation of SREBP-1c may be associated with the development of burned-out NASH through decreasing quantification of SREBP-1c positive hepatocyte nuclei and increasing mature SREBP-1c levels by immunoblot analysis (Nagaya et al., 2010). The study demonstrated that hepatic expression of SREBP-1c is increased in simple steatosis but gradually decreases with fibrosis progression. Based on these findings, we can speculate that Phb1 is relevant to the downregulation of hepatic expression levels of SREBPs and Insr, which control lipid metabolism and metabolic homeostasis by insulin. However, SREBPs were not predicted as upstream regulators between Phb1^−/−^ and Phb1^+/−^ (data not shown); further study is necessary to investigate the hepatic expression level of Insr and SREBPs in our liver disease model for elucidating contribution by gradual depletion of Phb1 on Insr-related insulin resistance and pathogenesis of liver injuries.

Peroxisome proliferator-activated receptor alpha (Pparα) was predicted as an increased upstream regulator in liver-specific Phb1^+/−^ compared with WT (*z*-score = 2.16) (Table 4). PPARα is a key regulator of lipid oxidation, which regulates genes involved in lipid and glucose metabolism and inflammation in the liver (Li and Palinski, 2006). PPARα agonists, such as fenofibrate and bezafibrate, are widely used to treat dyslipidemia, preventing hepatic steatosis and improving insulin sensitivity (Chou et al., 2002; Harano et al., 2006). We expected that if Pparα is an upstream regulator, it will decrease its function by Phb1 deficiency. Because our previous study showed that the *Ppar*γ mRNA expression level, one of the markers of hepatic steatosis, was increased in normal murine hepatocytes transfected with si*Phb1*, which mimics liver-specific Phb1^−/−^ (unpublished data, under review). On the other hand, our current study depicted that a slight decrease in Phb1 may attribute to increase Pparα. This trend was consistent with SREBPs as other upstream regulators. In a previous study on a patient with liver diseases, hepatic expression of SREBP-1c was increased in simple steatosis (SS) but gradually decreased in mild NASH and advanced NASH. Likewise, the hepatic mRNA level of *PPAR*α was significantly increased in SS and decreased in the mild NASH and advanced NASH (Nagaya et al., 2010). Taken together, we can infer that Phb1 deficiency at the early stage reflects a defective mechanism *via* Pparα against abnormal lipogenesis and/or lipid catabolism. But dramatic depletion of Phb1 is no capable of preventing impaired lipid metabolism and rapidly exacerbates liver injuries.

## Conclusions

In summary, our study demonstrated that the degree of depletion in Phb1 reflects different physiological responses. We characterized that Foxm1 and Timp1 can provide a basis for investigating a molecular mechanism on the increased susceptibility of liver injuries by Phb1 deficiency. Additionally, SREBPs and Pparα may be key genes to elucidate the pathogenesis of liver diseases relevant to Phb1 deficiency in terms of lipid metabolism. Taken together, these insights may lead to the establishment of novel therapeutic strategies against liver diseases containing liver cancer.

## Data Availability Statement

The data generated in this study can be accessed from NCBI using the accession numbers of the project and samples PRJNA738620, SAMN19735929.

## Ethics Statement

The animal study was reviewed and approved by Cedars-Sinai Medical Center IACUC (The IACUC protocol number is 9370).

## Author Contributions

KL performed pathway analysis and experiments and wrote the manuscript. HY performed the experiments. SS analyzed the data. BK performed RNA-seq analysis, data analysis, manuscript review, and pathway analysis. JL and S-HL performed RNA expression test. KK was responsible for mouse experiments, tissue collection, funding, designing and supervising the study, and revising the manuscript. All authors contributed to the article and approved the submitted version.

## Conflict of Interest

The authors declare that the research was conducted in the absence of any commercial or financial relationships that could be construed as a potential conflict of interest.

## Publisher's Note

All claims expressed in this article are solely those of the authors and do not necessarily represent those of their affiliated organizations, or those of the publisher, the editors and the reviewers. Any product that may be evaluated in this article, or claim that may be made by its manufacturer, is not guaranteed or endorsed by the publisher.

## References

[B1] Ahmed S. B. Prigent S. A. (2014). A nuclear export signal and oxidative stress regulate ShcD subcellular localisation: a potential role for ShcD in the nucleus. Cell. Signal. 26, 32–40. 10.1016/j.cellsig.2013.09.003 24036217

[B2] Aoki D. Oda Y. Hattori S. Taguchi K.-i. Ohishi Y. Basaki Y.. (2009). Overexpression of class III β-tubulin predicts good response to taxane-based chemotherapy in ovarian clear cell adenocarcinoma. Clin. Cancer Res. 15, 1473–1480. 10.1158/1078-0432.CCR-08-1274 19228748

[B3] Azzout-Marniche D. Bécard D. Guichard C. Foretz M. Ferré P. Foufelle. (2000). Insulin effects on sterol regulatory-element-binding protein-1c (SREBP-1c) transcriptional activity in rat hepatocytes. Biochem. J. 350, 389–393. 10.1042/bj3500389 10947952 PMC1221265

[B4] Barroso-González J. García-Expósito L. Hoang S. M. Lynskey M. L. Roncaioli J. L. Ghosh A.. (2019). RAD51AP1 is an essential mediator of alternative lengthening of telomeres. Mol. Cell 76, 11–26.e17. 10.1016/j.molcel.2019.06.043 31400850 PMC6778027

[B5] Benassi B. Flavin R. Marchionni L. Zanata S. Pan Y. Chowdhury D.. (2012). MYC is activated by USP2a-mediated modulation of microRNAs in prostate cancer. Cancer Discov. 2, 236–247. 10.1158/2159-8290.CD-11-0219 22585994 PMC3523361

[B6] Benjamin A. M. Nichols M. Burke T. W. Ginsburg G. S. Lucas J. E. (2014). Comparing reference-based RNA-Seq mapping methods for non-human primate data. BMC Genom. 15, 1–14. 10.1186/1471-2164-15-570 25001289 PMC4112205

[B7] Bolcun-Filas E. Bannister L. A. Barash A. Schimenti K. J. Hartford S. A. Eppig J. J.. (2011). A-MYB (MYBL1) transcription factor is a master regulator of male meiosis. Development 138, 3319–3330. 10.1242/dev.067645 21750041 PMC3133921

[B8] Bray F. Ferlay J. Soerjomataram I. Siegel R. L. Torre L. A. Jemal A. (2018). Global cancer statistics 2018: GLOBOCAN estimates of incidence and mortality worldwide for 36 cancers in 185 countries. Cancer J. Clin. 68, 394–424. 10.3322/caac.21492 30207593

[B9] Brown M. S. Goldstein J. L. (1997). The SREBP pathway: regulation of cholesterol metabolism by proteolysis of a membrane-bound transcription factor. Cell 89, 331–340. 10.1016/S0092-8674(00)80213-5 9150132

[B10] Calvisi D. F. Wang C. Ho C. Ladu S. Lee S. A. Mattu S.. (2011). Increased lipogenesis, induced by AKT-mTORC1-RPS6 signaling, promotes development of human hepatocellular carcinoma. Gastroenterology 140, 1071–1083.e1075. 10.1053/j.gastro.2010.12.006 21147110 PMC3057329

[B11] Chen G. Liang G. Ou J. Goldstein J. L. Brown M. S. (2004). Central role for liver X receptor in insulin-mediated activation of Srebp-1c transcription and stimulation of fatty acid synthesis in liver. Proc. Natl. Acad. Sci. 101, 11245–11250. 10.1073/pnas.0404297101 15266058 PMC509189

[B12] Chou C. J. Haluzik M. Gregory C. Dietz K. R. Vinson C. Gavrilova O.. (2002). WY14, 643, a peroxisome proliferator-activated receptor α (PPARα) agonist, improves hepatic and muscle steatosis and reverses insulin resistance in lipoatrophic A-ZIP/F-1 mice. J. Biol. Chem. 277, 24484–24489. 10.1074/jbc.M202449200 11994294

[B13] Cong S. Li Z. Yu L. Liu Y. Hu Y. Bi Y.. (2021). Integrative proteomic and lipidomic analysis of Kaili Sour Soup-mediated attenuation of high-fat diet-induced nonalcoholic fatty liver disease in a rat model. Nutr. Metabol. 18, 1–12. 10.1186/s12986-021-00553-4 33691721 PMC7945315

[B14] De Ingeniis J. Ratnikov B. Richardson A. D. Scott D. A. Aza-Blanc P. De S. K.. (2012). Functional specialization in proline biosynthesis of melanoma. PLoS ONE 7:e45190. 10.1371/journal.pone.0045190 23024808 PMC3443215

[B15] De Souza A. T. Dai X. Spencer A. G. Reppen T. Menzie A. Roesch P. L.. (2006). Transcriptional and phenotypic comparisons of Ppara knockout and siRNA knockdown mice. Nucl. Acids Res. 34, 4486–4494. 10.1093/nar/gkl609 16945951 PMC1636368

[B16] El-Brolosy M. A. Stainier D. Y. (2017). Genetic compensation: a phenomenon in search of mechanisms. PLoS Genet. 13:e1006780. 10.1371/journal.pgen.1006780 28704371 PMC5509088

[B17] Esmaeili M. Mirzaahmadi S. Asaadi Tehrani G. (2018). Evaluation of additional ABCC12 gene expression character in breast cancer samples using formal diagnostic profile. Gene Cell Tissue 5:e84392. 10.5812/gct.84392

[B18] Fusaro G. Dasgupta P. Rastogi S. Joshi B. Chellappan S. (2003). Prohibitin induces the transcriptional activity of p53 and is exported from the nucleus upon apoptotic signaling. J. Biol. Chem. 278, 47853–47861. 10.1074/jbc.M305171200 14500729

[B19] Ge Q. Feng F. Liu L. Chen L. Lv P. Ma S.. (2020). RNA-Seq analysis of the pathogenesis of STZ-induced male diabetic mouse liver. J. Diabet. Complications 34:107444. 10.1016/j.jdiacomp.2019.107444 31757765

[B20] Guo Y. Hu B. Huang H. Tsung A. Gaikwad N. W. Xu M.. (2015). Estrogen sulfotransferase is an oxidative stress-responsive gene that gender-specifically affects liver ischemia/reperfusion injury. J. Biol. Chem. 290, 14754–14764. 10.1074/jbc.M115.642124 25922074 PMC4505540

[B21] Gustafsson Å. B. Tsai J. G. Logue S. E. Crow M. T. Gottlieb R. A. (2004). Apoptosis repressor with caspase recruitment domain protects against cell death by interfering with Bax activation. J. Biol. Chem. 279, 21233–21238. 10.1074/jbc.M400695200 15004034

[B22] Haimerl F. Erhardt A. Sass G. Tiegs G. (2009). Down-regulation of the de-ubiquitinating enzyme ubiquitin-specific protease 2 contributes to tumor necrosis factor-α-induced hepatocyte survival. J. Biol. Chem. 284, 495–504. 10.1074/jbc.M803533200 19001362

[B23] Han J. Wang F. Lan Y. Wang J. Nie C. Liang Y.. (2019). KIFC1 regulated by miR-532-3p promotes epithelial-to-mesenchymal transition and metastasis of hepatocellular carcinoma *via* gankyrin/AKT signaling. Oncogene 38, 406–420. 10.1038/s41388-018-0440-8 30115976 PMC6336682

[B24] Harano Y. Yasui K. Toyama T. Nakajima T. Mitsuyoshi H. Mimani M.. (2006). Fenofibrate, a peroxisome proliferator-activated receptor α agonist, reduces hepatic steatosis and lipid peroxidation in fatty liver Shionogi mice with hereditary fatty liver. Liver Int. 26, 613–620. 10.1111/j.1478-3231.2006.01265.x 16762007

[B25] Hatanaka F. Takumi T. (2017). CHRONO integrates behavioral stress and epigenetic control of metabolism. Ann. Med. 49, 352–356. 28010116 10.1080/07853890.2016.1276301

[B26] Haupt Y. Maya R. Kazaz A. Oren M. (1997). Mdm2 promotes the rapid degradation of p53. Nature 387, 296–299. 10.1038/387296a0 9153395

[B27] Hegarty B. D. Bobard A. Hainault I. Ferré P. Bossard P. Foufelle F. (2005). Distinct roles of insulin and liver X receptor in the induction and cleavage of sterol regulatory elementbinding protein-1c. Proc. Natl. Acad. Sci. U. S. A. 102, 791–796. 10.1073/pnas.0405067102 15637161 PMC545517

[B28] Heo G. Ko K. S. (2019). Long-term feeding of soy protein attenuates choline deficient-induced adverse effects in wild type mice and prohibitin 1 deficient mice response more sensitively. Prev. Nutr. Food Sci. 24:32. 10.3746/pnf.2019.24.1.32 31008094 PMC6456240

[B29] Honda R. Yasuda H. (2000). Activity of MDM2, a ubiquitin ligase, toward p53 or itself is dependent on the RING finger domain of the ligase. Oncogene 19, 1473–1476. 10.1038/sj.onc.1203464 10723139

[B30] Hu C. Liu D. Zhang Y. Lou G. Huang G. Chen B.. (2014). LXRα-mediated downregulation of FOXM1 suppresses the proliferation of hepatocellular carcinoma cells. Oncogene 33, 2888–2897. 10.1038/onc.2013.250 23812424

[B31] Jeon T.-I. Osborne T. F. (2012). SREBPs: metabolic integrators in physiology and metabolism. Trends Endocrinol. Metabol. 23, 65–72. 10.1016/j.tem.2011.10.004 22154484 PMC3273665

[B32] Jung S. Park J. Ko K. S. (2020). Lipopolysaccharide-induced innate immune responses are exacerbated by Prohibitin 1 deficiency and mitigated by S-adenosylmethionine in murine macrophages. PLoS ONE 15:e0241224. 10.1371/journal.pone.0241224 33175859 PMC7657527

[B33] Kakino S. Ohki T. Nakayama H. Yuan X. Otabe S. Hashinaga T.. (2018). Pivotal role of TNF-α in the development and progression of nonalcoholic fatty liver disease in a murine model. Hormone Metabolic Res. 50, 80–87. 10.1055/s-0043-118666 28922680

[B34] Kitamura H. Hashimoto M. (2021). USP2-related cellular signaling and consequent pathophysiological outcomes. Int. J. Mol. Sci. 22:1209. 10.3390/ijms22031209 33530560 PMC7865608

[B35] Knebel B. Hartwig S. Jacob S. Kettel U. Schiller M. Passlack W.. (2018). Inactivation of SREBP-1a phosphorylation prevents fatty liver disease in mice: identification of related signaling pathways by gene expression profiles in liver and proteomes of peroxisomes. Int. J. Mol. Sci. 19:980. 10.3390/ijms19040980 29587401 PMC5979561

[B36] Ko K. S. Tomasi M. L. Iglesias-Ara A. French B. A. French S. W. Ramani K.. (2010). Liver-specific deletion of prohibitin 1 results in spontaneous liver injury, fibrosis, and hepatocellular carcinoma in mice. Hepatology 52, 2096–2108. 10.1002/hep.23919 20890892 PMC3005187

[B37] Kong B.-W. Hudson N. Seo D. Lee S. Khatri B. Lassiter K.. (2017). RNA sequencing for global gene expression associated with muscle growth in a single male modern broiler line compared to a foundational Barred Plymouth Rock chicken line. BMC Genom. 18, 1–19. 10.1186/s12864-016-3471-y 28086790 PMC5237145

[B38] Kopanja D. Pandey A. Kiefer M. Wang Z. Chandan N. Carr J. R.. (2015). Essential roles of FoxM1 in Ras-induced liver cancer progression and in cancer cells with stem cell features. J. Hepatol. 63, 429–436. 10.1016/j.jhep.2015.03.023 25828473 PMC4508215

[B39] Lee J. Kong B. Lee S.-H. (2020). Patchouli alcohol, a compound from Pogostemoncablin, inhibits obesity. J. Med. Food 23, 326–334. 10.1089/jmf.2019.0182 31750759

[B40] Lee J.-K. Wang J. Sa J. K. Ladewig E. Lee H.-O. Lee I.-H.. (2017). Spatiotemporal genomic architecture informs precision oncology in glioblastoma. Nat. Genet. 49, 594–599. 10.1038/ng.3806 28263318 PMC5627771

[B41] Li A. C. Palinski W. (2006). Peroxisome proliferator-activated receptors: how their effects on macrophages can lead to the development of a new drug therapy against atherosclerosis. Annu. Rev. Pharmacol. Toxicol. 46, 1–39. 10.1146/annurev.pharmtox.46.120604.141247 16402897

[B42] Li L. Falany C. N. (2007). Elevated hepatic SULT1E1 activity in mouse models of cystic fibrosis alters the regulation of estrogen responsive proteins. J. Cystic Fibr. 6, 23–30. 10.1016/j.jcf.2006.05.001 16798114

[B43] Li L. He D. Wilborn T. W. Falany J. L. Falany C. N. (2009). Increased SULT1E1 activity in HepG2 hepatocytes decreases growth hormone stimulation of STAT5b phosphorylation. Steroids 74, 20–29. 10.1016/j.steroids.2008.09.002 18831980 PMC2633718

[B44] Li Y. Lu L. Tu J. Zhang J. Xiong T. Fan W.. (2020). Reciprocal regulation between forkhead box M1/NF-κB and methionine adenosyltransferase 1A drives liver cancer. Hepatology 72, 1682–1700. 10.1002/hep.31196 32080887 PMC7442711

[B45] Loayza-Puch F. Rooijers K. Buil L. C. Zijlstra J. Vrielink J. F. O. Lopes R.. (2016). Tumour-specific proline vulnerability uncovered by differential ribosome codon reading. Nature 530, 490–494. 10.1038/nature16982 26878238

[B46] Lounis M. A. Escoula Q. Veillette C. Bergeron K.-F. Ntambi J. M. Mounier C. (2016). SCD1 deficiency protects mice against ethanol-induced liver injury. Biochim. Biophys. Acta 1861, 1662–1670. 10.1016/j.bbalip.2016.07.012 27477676

[B47] Matsushita N. Hassanein M. T. Martinez-Clemente M. Lazaro R. French S. W. Xie W.. (2017). Gender difference in NASH susceptibility: roles of hepatocyte Ikkβ and Sult1e1. PLoS ONE 12:e0181052. 10.1371/journal.pone.0181052 28797077 PMC5552280

[B48] Miao J. Haas J. T. Manthena P. Wang Y. Zhao E. Vaitheesvaran B.. (2014). Hepatic insulin receptor deficiency impairs the SREBP-2 response to feeding and statins. J. Lip. Res. 55, 659–667. 10.1194/jlr.M043711 24516236 PMC3966700

[B49] Michael M. D. Kulkarni R. N. Postic C. Previs S. F. Shulman G. I. Magnuson M. A.. (2000). Loss of insulin signaling in hepatocytes leads to severe insulin resistance and progressive hepatic dysfunction. Mol. Cell 6, 87–97. 10.1016/S1097-2765(05)00015-8 10949030

[B50] Mishra S. Murphy L. C. Murphy L. J. (2006). The Prohibitins: emerging roles in diverse functions. J. Cell. Mol. Med. 10, 353–363. 10.1111/j.1582-4934.2006.tb00404.x 16796804 PMC3933126

[B51] Moslehi A. Hamidi-Zad Z. (2018). Role of SREBPs in liver diseases: a mini-review. J. Clin. Transl. Hepatol. 6:332. 10.14218/JCTH.2017.00061 30271747 PMC6160306

[B52] Muranaka H. Hayashi A. Minami K. Kitajima S. Kohno S. Nishimoto Y.. (2017). A distinct function of the retinoblastoma protein in the control of lipid composition identified by lipidomic profiling. Oncogenesis 6:e350. 10.1038/oncsis.2017.51 28650445 PMC5519198

[B53] Nagaya T. Tanaka N. Suzuki T. Sano K. Horiuchi A. Komatsu M.. (2010). Down-regulation of SREBP-1c is associated with the development of burned-out NASH. J. Hepatol. 53, 724–731. 10.1016/j.jhep.2010.04.033 20655124

[B54] Nijtmans L. G. de Jong L. Sanz M. A. Coates P. J. Berden J. A. Back J. W.. (2000). Prohibitins act as a membrane-bound chaperone for the stabilization of mitochondrial proteins. EMBO J. 19, 2444–2451. 10.1093/emboj/19.11.2444 10835343 PMC212747

[B55] Ramani K. Mavila N. Ko K. S. Mato J. M. Lu S. C. (2016). Prohibitin 1 regulates the H19-Igf2 axis and proliferation in hepatocytes. J. Biol. Chem. 291, 24148–24159. 10.1074/jbc.M116.744045 27687727 PMC5104939

[B56] Rossi A. Kontarakis Z. Gerri C. Nolte H. Hölper S. Krüger M.. (2015). Genetic compensation induced by deleterious mutations but not gene knockdowns. Nature 524, 230–233. 10.1038/nature14580 26168398

[B57] Saavedra-García P. Nichols K. Mahmud Z. Fan L. Y.-N. Lam E. W. (2018). Unravelling the role of fatty acid metabolism in cancer through the FOXO3-FOXM1 axis. Mol. Cell. Endocrinol. 462, 82–92. 10.1016/j.mce.2017.01.012 28087388

[B58] Samuel V. T. Shulman G. I. (2012). Mechanisms for insulin resistance: common threads and missing links. Cell 148, 852–871. 10.1016/j.cell.2012.02.017 22385956 PMC3294420

[B59] Savarin C. Bergmann C. C. Hinton D. R. Stohlman S. A. (2013). MMP-independent role of TIMP-1 at the blood brain barrier during viral encephalomyelitis. ASN Neuro 5:AN20130033. 10.1042/AN20130033 24156369 PMC3840398

[B60] Shimomura I. Bashmakov Y. Ikemoto S. Horton J. D. Brown M. S. Goldstein J. L. (1999). Insulin selectively increases SREBP-1c mRNA in the livers of rats with streptozotocin-induced diabetes. Proc. Natl. Acad. Sci. U. S. A. 96, 13656–13661. 10.1073/pnas.96.24.13656 10570128 PMC24120

[B61] Smalling R. L. Delker D. A. Zhang Y. Nieto N. Mcguiness M. S. Liu S.. (2013). Genome-wide transcriptome analysis identifies novel gene signatures implicated in human chronic liver disease. Am. J. Physiol. Gastrointestinal Liver Physiol. 305, G364–G374. 10.1152/ajpgi.00077.2013 23812039 PMC3761248

[B62] Song B. Zhang H. Zhang S. (2018). Toll-like receptor 2 mediates deposition of collagen I in adipose tissue of high fat diet-induced obese mice. Mol. Med. Rep. 17, 5958–5963. 10.3892/mmr.2018.8590 29436650

[B63] Song W. C. (2001). Biochemistry and reproductive endocrinology of estrogen sulfotransferase. Ann. New York Acad. Sci. 948, 43–50. 11795394 10.1111/j.1749-6632.2001.tb03985.x

[B64] Souri M. Osaki T. Ichinose A. (2015). The non-catalytic B subunit of coagulation factor XIII accelerates fibrin cross-linking. J. Biol. Chem. 290, 12027–12039. 10.1074/jbc.M114.608570 25809477 PMC4424339

[B65] Stevenson L. F. Sparks A. Allende-Vega N. Xirodimas D. P. Lane D. P. Saville M. K. (2007). The deubiquitinating enzyme USP2a regulates the p53 pathway by targeting Mdm2. EMBO J. 26, 976–986. 10.1038/sj.emboj.7601567 17290220 PMC1852834

[B66] Stuebner S. Faus-Kessler T. Fischer T. Wurst W. Prakash N. (2010). Fzd3 and Fzd6 deficiency results in a severe midbrain morphogenesis defect. Dev. Dyn. 239, 246–260. 10.1002/dvdy.22127 19842188

[B67] Sun H.-C. Li M. Lu J.-L. Yan D.-W. Zhou C.-Z. Fan J.-W.. (2011). Overexpression of Forkhead box M1 protein associates with aggressive tumor features and poor prognosis of hepatocellular carcinoma. Oncol. Rep. 25, 1533–1539. 10.3892/or.2011.1230 21431285

[B68] Sun Q. Zhao Y. Yang Y. Yang X. Li M. Xu X.. (2017). Loss of the clock protein PER2 shortens the erythrocyte life span in mice. J. Biol. Chem. 292, 12679–12690. 10.1074/jbc.M117.783985 28607147 PMC5535041

[B69] Talukdar I. Szeszel-Fedorowicz W. Salati L. M. (2005). Arachidonic acid inhibits the insulin induction of glucose-6-phosphate dehydrogenase via p38 MAP kinase. J. Biol. Chem. 280, 40660–40667. 10.1074/jbc.M505531200 16210322

[B70] Tang H. Goldberg E. (2012). A-MYB (MYBL1) stimulates murine testis-specific Ldhc expression *via* the cAMP-responsive element (CRE) site. Biol. Reprod. 86, 30, 31–38. 10.1095/biolreprod.111.095661 21998171 PMC3290662

[B71] Teh M.-T. (2012). FOXM1 coming of age: time for translation into clinical benefits? Front. Oncol. 2:146. 10.3389/fonc.2012.00146 23087907 PMC3471356

[B72] Thiele N. D. Wirth J. W. Steins D. Koop A. C. Ittrich H. Lohse A. W.. (2017). TIMP-1 is upregulated, but not essential in hepatic fibrogenesis and carcinogenesis in mice. Sci. Rep. 7, 1–9. 10.1038/s41598-017-00671-1 28386095 PMC5428806

[B73] Tomita K. Tamiya G. Ando S. Ohsumi K. Chiyo T. Mizutani A.. (2006). Tumour necrosis factor α signalling through activation of Kupffer cells plays an essential role in liver fibrosis of non-alcoholic steatohepatitis in mice. Gut 55, 415–424. 10.1136/gut.2005.071118 16174657 PMC1856073

[B74] Urlep Ž. Lorbek G. Perše M. Jeruc J. Juvan P. Matz-Soja M.. (2017). Disrupting hepatocyte Cyp51 from cholesterol synthesis leads to progressive liver injury in the developing mouse and decreases RORC signalling. Sci. Rep. 7, 1–13. 10.1038/srep40775 28098217 PMC5241696

[B75] Wang H. Lafdil F. Wang L. Yin S. Feng D. Gao B. (2011). Tissue inhibitor of metalloproteinase 1 (TIMP-1) deficiency exacerbates carbon tetrachloride-induced liver injury and fibrosis in mice: involvement of hepatocyte STAT3 in TIMP-1 production. Cell Biosci. 1, 1–10. 10.1186/2045-3701-1-14 21711826 PMC3125204

[B76] Wang L. Feng Z. Wang X. Wang X. Zhang X. (2010). DEGseq: an R package for identifying differentially expressed genes from RNA-seq data. Bioinformatics 26, 136–138. 10.1093/bioinformatics/btp612 19855105

[B77] Wang L. Zhang C.-g. Jia Y.-l. Hu L. (2020). Tissue Inhibitor of Metalloprotease-1 (TIMP-1) Regulates Adipogenesis of Adipose-derived Stem Cells (ASCs) *via* the Wnt Signaling Pathway in an MMP-independent Manner. Curr. Med. Sci. 40, 989–996. 10.1007/s11596-020-2265-2 33123912

[B78] Wang S. Fusaro G. Padmanabhan J. Chellappan S. P. (2002a). Prohibitin co-localizes with Rb in the nucleus and recruits N-CoR and HDAC1 for transcriptional repression. Oncogene 21, 8388–8396. 10.1038/sj.onc.1205944 12466959

[B79] Wang S. Zhang B. Faller D. V. (2002b). Prohibitin requires Brg-1 and Brm for the repression of E2F and cell growth. EMBO J. 21, 3019–3028. 10.1093/emboj/cdf302 12065415 PMC126057

[B80] Wang S. Zhang B. Faller D. V. (2004). BRG1/BRM and prohibitin are required for growth suppression by estrogen antagonists. EMBO J. 23, 2293–2303. 10.1038/sj.emboj.7600231 15141164 PMC419909

[B81] Wei Y.-L. Yang W.-X. (2019). Kinesin-14 motor protein KIFC1 participates in DNA synthesis and chromatin maintenance. Cell Death Dis. 10, 1–14. 10.1038/s41419-019-1619-9 31127080 PMC6534603

[B82] Wills M. K. Tong J. Tremblay S. L. Moran M. F. Jones N. (2014). The ShcD signaling adaptor facilitates ligand-independent phosphorylation of the EGF receptor. Mol. Biol. Cell 25, 739–752. 10.1091/mbc.e13-08-0434 24430869 PMC3952845

[B83] Wu L. Nam Y.-J. Kung G. Crow M. T. Kitsis R. N. (2010). Induction of the apoptosis inhibitor ARC by Ras in human cancers. J. Biol. Chem. 285, 19235–19245. 10.1074/jbc.M110.114892 20392691 PMC2885202

[B84] Wu Y. Wang H. Qiao L. Jin X. Dong H. Wang Y. (2019). Silencing of RAD51AP1 suppresses epithelial–mesenchymal transition and metastasis in non-small cell lung cancer. Thoracic Cancer 10, 1748–1763. 10.1111/1759-7714.13124 31317661 PMC6718026

[B85] Xiao X. Song B.-L. (2013). SREBP: a novel therapeutic target. Actabiochim. BiophysicaSinica 45, 2–10. 10.1093/abbs/gms112 23257291

[B86] Xu Y. Lin X. Xu J. Jing H. Qin Y. Li Y. (2018). SULT1E1 inhibits cell proliferation and invasion by activating PPARγ in breast cancer. J. Cancer 9:1078. 10.7150/jca.23596 29581787 PMC5868175

[B87] Yan W. Jiang M. Zheng J. (2020). Identification of key pathways and differentially expressed genes in bronchopulmonary dysplasia using bioinformatics analysis. Biotechnol. Lett. 42, 2569–2580. 10.1007/s10529-020-02986-y 32803430

[B88] Yang J. Li B. He Q.-Y. (2018). Significance of prohibitin domain family in tumorigenesis and its implication in cancer diagnosis and treatment. Cell Death Dis. 9, 1–10. 10.1038/s41419-018-0661-3 29784973 PMC5962566

[B89] Ylisirniö S. Höyhtyä M. Turpeenniemi-Hujanen T. (2000). Serum matrix metalloproteinases-2,-9 and tissue inhibitors of metalloproteinases-1,-2 in lung cancer–TIMP-1 as a prognostic marker. Anticancer Res. 20, 1311–1316. 10810441

[B90] Yoshiji H. Kuriyama S. Miyamoto Y. Thorgeirsson U. P. Gomez D. E. Kawata M.. (2000). Tissue inhibitor of metalloproteinases-1 promotes liver fibrosis development in a transgenic mouse model. Hepatology 32, 1248–1254. 10.1053/jhep.2000.20521 11093731

[B91] Young M.-J. Hsu K.-C. Lin T. E. Chang W.-C. Hung J.-J. (2019). The role of ubiquitin-specific peptidases in cancer progression. J. Biomed. Sci. 26, 1–14. 10.1186/s12929-019-0522-0 31133011 PMC6537419

[B92] Young R. Passet B. Vilotte M. Cribiu E.P. Béringue V. Le Provost F.. (2009). The prion or the related Shadoo protein is required for early mouse embryogenesis. FEBS letters 583, 3296–3300. 19766638 10.1016/j.febslet.2009.09.027

[B93] Yukawa N. Yoshikawa T. Akaike M. Sugimasa Y. Rino Y. Masuda M.. (2007). Impact of plasma tissue inhibitor of matrix metalloproteinase-1 on long-term survival in patients with colorectal cancer. Oncology 72, 205–208. 10.1159/000112827 18160809

[B94] Zeng Z. He W. Jia Z. Hao S. (2017). Lycopene improves insulin sensitivity through inhibition of STAT3/Srebp-1c-mediated lipid accumulation and inflammation in mice fed a high-fat diet. Exp. Clin. Endocrinol. Diabetes 125, 610–617. 10.1055/s-0043-101919 28472825

