# Supplementary material for: RNA-Seq Reveals Different Gene Expression in Liver-Specific Prohibitin 1 Knock-Out Mice
Source: Front Physiol. 2021 Sep 3;12:717911. doi: 10.3389/fphys.2021.717911 (PMC8446661; doi:10.3389/fphys.2021.717911)
Supplement: Supplementary file 1 [file Data_Sheet_1.docx]

**Supplementary material 1. Primer sequence for RT-PCR analysis.**

| Gene | Species | mRNA NO. | Primer | Sequence (5’→3’) |
| --- | --- | --- | --- | --- |
| *Foxm1* | Murine | NM_008021.4 | Forward | AGAGAAAAGGGAGGTGAGCC |
|  |  |  | Reverse | GAGTGGAGGAGACAGGCAAT |
| *Timp1* | Murine | NM_001044384.1 | Forward | TTCAAGGCTGTGGGAAAT |
|  |  |  | Reverse | CCACAGCCAGCACTATAGGT |
| *Usp2* | Murine | NM_016808.2 | Forward | CTCCACCTGAAGCGCTATA |
|  |  |  | Reverse | CCCCTGTCACAGTCCAGAAT |
| *Scd1* | Murine | NM_009127.4 | Forward | GCCTGTTCGTTAGCACCTTC |
|  |  |  | Reverse | ACGTGGTGAAGTTGATGTGC |
| *Sult1e1* | Murine | NM_023135.2 | Forward | GTTCCTGGAGAGAAAGCCCT |
|  |  |  | Reverse | TCTGCTGCTTGTAGTGCTCA |
| *Gapdh* | Murine | NM_001289726.1 | Forward | CAGGAGCGAGACCCCACTAACAT |
|  |  |  | Reverse | GTCAGATCCACGACGGACACATT |

**Supplementary material 2. Primer sequence for qRT-PCR analysis.**

| Gene | Species | mRNA NO. | Primer | Sequence (5’→3’) |
| --- | --- | --- | --- | --- |
| *Phb1* | Murine | NM_008831.4 | Forward | GTGGTGAACTCTGCTTTGTA |
|  |  |  | Reverse | CCAAGGGATGAGGAAATGAG |
| *Foxm1* | Murine | NM_008021.4 | Forward | AAGGGCTGTTGCCTTTAC |
|  |  |  | Reverse | AGGGCTCTCCACTTTGATA |
| *Timp1* | Murine | NM_001044384.1 | Forward | GGTGGGTGGATGAGTAATG |
|  |  |  | Reverse | GCTGCACAGTGGAGAATAA |
| *Usp2* | Murine | NM_016808.2 | Forward | CTCAGCTGCTTCCCTTTATC |
|  |  |  | Reverse | CATTTACCCACTTCCCTCTTT |
| *Scd1* | Murine | NM_009127.4 | Forward | TTAGCACCTTCTTGCGATAC |
|  |  |  | Reverse | CCCGGGATTGAATGTTCTT |
| *Sult1e1* | Murine | NM_023135.2 | Forward | GAGTTCCGTGGAGTTCTAATG |
|  |  |  | Reverse | GGTGGTACCAGATTTAGGATATG |
| *Actb* | Murine | NM_007393.5 | Forward | TGACGTTGACATCCGTAAAG |
|  |  |  | Reverse | AGGAGCCAGAGCAGTAAT |

**Supplementary material 3. The 78 DE genes in Phb1^+/-^ vs. WT and Phb1^-/-^ vs. WT.**

|  | Hetero vs WT | | KO vs. WT | |  |
| --- | --- | --- | --- | --- | --- |
| Name | Log_2_FC | FDR | Log_2_FC | FDR | Entrez Gene Name |
| Gm40368 | **1.3** | **0.039** | **2.5** | **0.000** | predicted gene, 40368 |
| Nol3 | **1.1** | **0.048** | **4.2** | **0.000** | nucleolar protein 3 |
| Snora17 | **1.0** | **0.019** | **2.2** | **0.000** | small nucleolar RNA, H/ACA box 17 |
| Gm40498 | **7.0** | **0.013** | **10.0** | **0.001** | predicted gene, 40498 |
| Gm27252 | **1.1** | **0.021** | **-1.2** | **0.015** | predicted gene 27252 |
| Gm38832 | **1.4** | **0.000** | **-1.2** | **0.001** | predicted gene, 38832 |
| Gm7429 | **1.3** | **0.024** | **1.7** | **0.004** | predicted pseudogene 7429 |
| Gm15510 | **1.3** | **0.015** | **1.2** | **0.024** | predicted gene 15510 |
| Gm35601 | **1.3** | **0.009** | -0.5 | 0.265 | predicted gene, 35601 |
| Sult1e1 | **2.7** | **0.004** | **2.4** | **0.009** | sulfotransferase family 1E member 1 |
| Usp2 | **1.3** | **0.009** | -0.6 | 0.239 | ubiquitin specific peptidase 2 |
| Ciart | **1.4** | **0.040** | 0.4 | 0.578 | circadian associated repressor of transcription |
| Gm39966 | **1.2** | **0.043** | 0.1 | 0.939 | predicted gene, 39966 |
| Hist1h2bn | **5.5** | **0.035** | 2.3 | 0.422 | histone cluster 1 H2B family member m |
| Per2 | **1.0** | **0.021** | 0.6 | 0.186 | period circadian regulator 2 |
| 6030419C18Rik | **-4.1** | **0.028** | **5.4** | **0.006** |  |
| Abcc12 | **-4.6** | **0.004** | **3.9** | **0.013** | ATP binding cassette subfamily C member 12 |
| Acta2 | **-1.9** | **0.022** | **1.7** | **0.038** | actin, alpha 2, smooth muscle, aorta |
| Adamts9 | **-1.9** | **0.001** | **1.8** | **0.001** | ADAM metallopeptidase with thrombospondin type 1 motif 9 |
| Amn | **-1.2** | **0.047** | **3.3** | **0.000** | amnion associated transmembrane protein |
| Atp4a | **-1.6** | **0.002** | **2.7** | **0.000** | ATPase H+/K+ transporting subunit alpha |
| Cib3 | **-1.4** | **0.021** | **1.8** | **0.005** | calcium and integrin binding family member 3 |
| Col1a1 | **-1.1** | **0.035** | **2.0** | **0.000** | collagen type I alpha 1 chain |
| Ddias | **-1.1** | **0.030** | **3.1** | **0.000** | DNA damage induced apoptosis suppressor |
| Dpt | **-1.1** | **0.006** | **1.1** | **0.005** | dermatopontin |
| Dusp5 | **-1.7** | **0.002** | **2.4** | **0.000** | dual specificity phosphatase 5 |
| F13a1 | **-4.1** | **0.011** | **4.6** | **0.005** | coagulation factor XIII A chain |
| Fabp5 | **-1.8** | **0.000** | **2.3** | **0.000** | fatty acid binding protein 5 |
| Foxm1 | **-1.6** | **0.008** | **3.0** | **0.000** | forkhead box M1 |
| Fzd3 | **-3.9** | **0.023** | **5.3** | **0.003** | frizzled class receptor 3 |
| Gmds | **-1.1** | **0.037** | **2.3** | **0.000** | GDP-mannose 4,6-dehydratase |
| Gsta1 | **-2.9** | **0.023** | **3.2** | **0.011** | glutathione S-transferase alpha 5 |
| Mybl1 | **-3.3** | **0.021** | **3.8** | **0.008** | MYB proto-oncogene like 1 |
| Nusap1 | **-1.6** | **0.024** | **2.6** | **0.001** | nucleolar and spindle associated protein 1 |
| Pafah1b3 | **-1.1** | **0.003** | **1.6** | **0.000** | platelet activating factor acetylhydrolase 1b catalytic subunit 3 |
| Pbk | **-1.3** | **0.015** | **4.0** | **0.000** | PDZ binding kinase |
| Plat | **-1.5** | **0.015** | **3.9** | **0.000** | plasminogen activator, tissue type |
| Prmt2 | **-1.2** | **0.019** | **2.4** | **0.000** | protein arginine methyltransferase 2 |
| Pycr1 | **-3.8** | **0.009** | **5.3** | **0.001** | pyrroline-5-carboxylate reductase 1 |
| Rad51ap1 | **-3.5** | **0.023** | **3.3** | **0.029** | RAD51 associated protein 1 |
| Sept5 | **-1.5** | **0.017** | **1.6** | **0.008** | septin 5 |
| Shc4 | **-4.5** | **0.007** | **5.1** | **0.003** | SHC adaptor protein 4 |
| Spag5 | **-1.2** | **0.024** | **2.9** | **0.000** | sperm associated antigen 5 |
| St8sia3 | **-1.0** | **0.050** | **2.3** | **0.000** | ST8 alpha-N-acetyl-neuraminide alpha-2,8-sialyltransferase 3 |
| Timp1 | **-4.4** | **0.012** | **6.4** | **0.001** | TIMP metallopeptidase inhibitor 1 |
| Troap | **-1.2** | **0.038** | **3.0** | **0.000** | trophinin associated protein |
| Tubb2b | **-4.8** | **0.015** | **7.6** | **0.001** | tubulin beta 2B class IIb |
| Asb2 | **-1.3** | **0.010** | -0.1 | 0.849 | ankyrin repeat and SOCS box containing 2 |
| Fam198a | **-1.7** | **0.048** | 1.6 | 0.062 | family with sequence similarity 198 member A |
| Fdps | **-1.8** | **0.008** | -0.5 | 0.505 | farnesyl diphosphate synthase |
| Gm8210 | **-2.4** | **0.033** | 1.0 | 0.439 | ribosomal protein L29 |
| Gm33459 | **-1.6** | **0.015** | 0.0 | 0.986 | predicted gene, 33459 |
| Ifi205 | **-1.0** | **0.022** | 0.6 | 0.216 | interferon gamma inducible protein 16 |
| Kifc5b | **-3.9** | **0.048** | 2.2 | 0.284 | kinesin family member C1 |
| Mvd | **-1.3** | **0.045** | 0.1 | 0.899 | mevalonate diphosphate decarboxylase |
| Pcsk9 | **-1.2** | **0.049** | 0.7 | 0.312 | proprotein convertase subtilisin/kexin type 9 |
| Slc34a2 | **-1.0** | **0.019** | 0.4 | 0.424 | solute carrier family 34 member 2 |
| Spsb4 | **-1.3** | **0.018** | 1.0 | 0.058 | splA/ryanodine receptor domain and SOCS box containing 4 |
| Sqle | **-2.1** | **0.002** | 0.4 | 0.559 | squalene epoxidase |
| Cyp51 | **-1.8** | **0.009** | -0.6 | 0.422 | cytochrome P450 family 51 subfamily A member 1 |
| Idi1 | **-1.9** | **0.023** | -0.6 | 0.519 | isopentenyl-diphosphate delta isomerase 1 |
| LOC108168990 | **-2.0** | **0.017** | -0.9 | 0.278 |  |
| Nsdhl | **-1.7** | **0.016** | -0.4 | 0.577 | NAD(P) dependent steroid dehydrogenase-like |
| AW549542 | **-1.1** | **0.043** | **-2.3** | **0.000** | expressed sequence AW549542 |
| Cyp2c69 | **-1.3** | **0.039** | **-6.7** | **0.000** | cytochrome P450, family 2, subfamily c, polypeptide 40 |
| Selenbp2 | **-1.7** | **0.042** | **-3.5** | **0.000** | selenium binding protein 1 |
| Slc22a28 | **-1.2** | **0.043** | **-4.0** | **0.000** | solute carrier family 22 member 25 |
| 2310015A16Rik | **-2.2** | **0.000** | **-2.1** | **0.000** | RIKEN cDNA 2310015A16 gene |
| Abca8a | **-1.0** | **0.003** | **-1.3** | **0.000** | ATP-binding cassette, sub-family A (ABC1), member 8a |
| Gm32643 | **-1.9** | **0.039** | **-2.5** | **0.006** | predicted gene, 32643 |
| Klk1b4 | **-1.3** | **0.000** | **-1.0** | **0.001** | kallikrein related peptidase 3 |
| Rdh11 | **-1.1** | **0.015** | -0.2 | 0.763 | retinol dehydrogenase 11 |
| Scd1 | **-1.4** | **0.026** | **-1.5** | **0.016** | stearoyl-CoA desaturase |
| Gm36193 | **-1.3** | **0.015** | -0.5 | 0.353 | predicted gene, 36193 |
| Msmo1 | **-1.5** | **0.013** | -1.1 | 0.063 | methylsterol monooxygenase 1 |
| Tgtp2 | **-1.3** | **0.019** | -0.6 | 0.275 | T cell specific GTPase 1 |
| Zbp1 | **-1.3** | **0.016** | -0.3 | 0.587 | Z-DNA binding protein 1 |
| Gdpd1 | **-1.0** | **0.003** | **2.6** | **0.000** | glycerophosphodiester phosphodiesterase domain containing 1 |

“Hetero” and “KO” in tables represents “Phb1^+/-^” and “Phb1^-/-^”, respectively. Log_2_FC with FDR < 0.05 between groups are shown in bold.


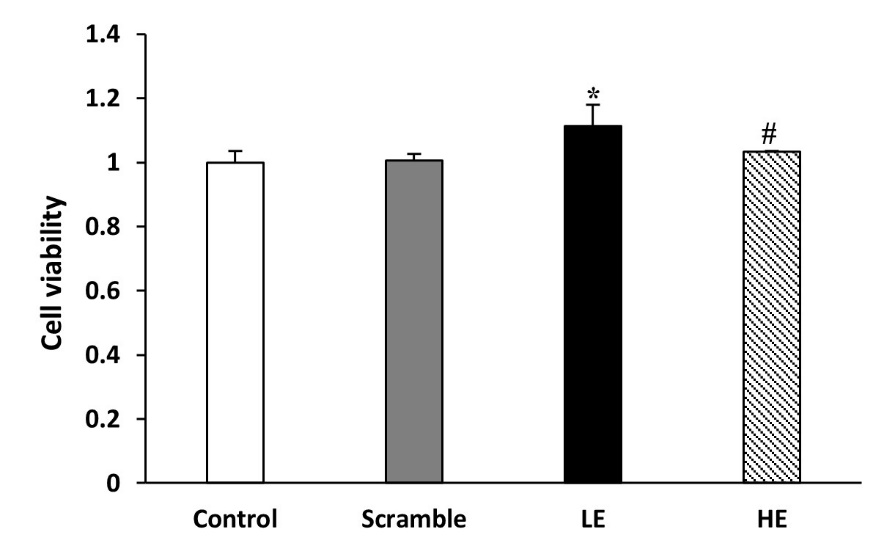
**Supplementary material 4. Cell viability in AML12 cells with transfected *Phb1* siRNA.**

“LE” means “low efficiency”, represented Phb1^+/-^, and “HE” means “high efficiency”, represented Phb1^-/-^. All data were expressed as means ± standard deviation. One-way ANOVA followed by Duncan post hoc test was performed and differences were considered statistically significant. * *p < 0.05* versus control. # *p < 0.05* versus low efficiency (LE).


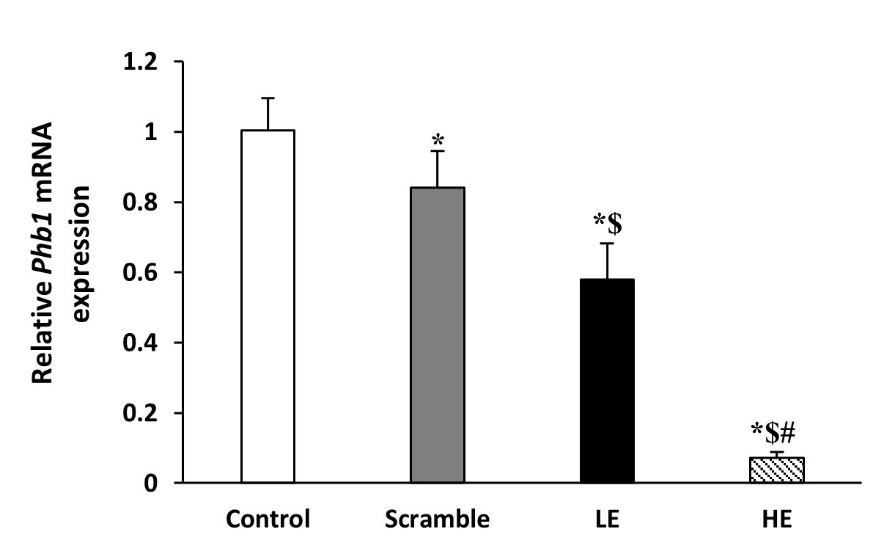
**Supplementary material 5. Relative Phb1 mRNA expression levels in AML12 cells with transfected *Phb1* siRNA.**

“LE” means “low efficiency”, represented Phb1^+/-^, and “HE” means “high efficiency”, represented Phb1^-/-^. All data were expressed as means ± standard deviation. One-way ANOVA followed by Duncan post hoc test was performed and differences were considered statistically significant. * *p < 0.05* versus control. # *p < 0.05* versus low efficiency (LE). $ *p < 0.05* versus scramble.

**Supplementary material 6. Top 10 Disease and Bio Functions between Phb1^+/-^ and WT.**

| Categories | Diseases or Functions Annotation | p-Value | Molecules |
| --- | --- | --- | --- |
| Gastrointestinal Disease, Hepatic System Disease, Organismal Injury and Abnormalities | Fibrosis of liver | 0.000 | SCD,ACTA2,COL1A1,TIMP1 |
| Cancer, Organismal Injury and Abnormalities | Secondary tumor | 0.001 | PBK,ACTA2,PLAT,TIMP1 |
| Cardiovascular System Development and Function | Development of vasculature | 0.002 | FOXM1,COL1A1,TIMP1 |
| Cardiovascular Disease, Cardiovascular System Development and Function, Digestive System Development and Function, Gastrointestinal Disease, Hepatic System Development and Function, Hepatic System Disease, Organ Morphology, Organismal Development, Organismal Injury and Abnormalities, Tissue Morphology | Abnormal morphology of hepatic vein | 0.004 | FOXM1 |
| Cardiovascular System Development and Function, Digestive System Development and Function, Gastrointestinal Disease, Hepatic System Development and Function, Hepatic System Disease, Organ Morphology, Organismal Development, Organismal Injury and Abnormalities, Tissue Morphology | Abnormal morphology of intrahepatic bile duct | 0.004 | FOXM1 |
| Lipid Metabolism, Molecular Transport, Small Molecule Biochemistry | Accumulation of oleic acid | 0.004 | SCD |
| Lipid Metabolism, Molecular Transport, Small Molecule Biochemistry | Accumulation of palmitoleic acid | 0.004 | SCD |
| Cell-To-Cell Signaling and Interaction, Cellular Assembly and Organization | Association of plasma membrane | 0.004 | SCD |
| Cancer, Gastrointestinal Disease, Hepatic System Disease, Organismal Injury and Abnormalities | Cancer of hepatocytes | 0.004 | TIMP1 |
| Tissue Development | Deposition of extracellular matrix | 0.004 | TIMP1 |
| Carbohydrate Metabolism, Small Molecule Biochemistry | Incorporation of glycerol | 0.004 | SCD |
| Lipid Metabolism, Molecular Transport, Small Molecule Biochemistry | Secretion of fatty acid | 0.004 | SCD |
| Cancer, Gastrointestinal Disease, Organismal Injury and Abnormalities | Metastatic colorectal cancer | 0.006 | PBK,ACTA2,PLAT |
| Lipid Metabolism, Molecular Transport, Small Molecule Biochemistry | Quantity of saturated fatty acid | 0.007 | SCD |
| Tissue Morphology | Degradation of extracellular matrix | 0.011 | TIMP1 |
| Lipid Metabolism, Molecular Transport, Small Molecule Biochemistry | Quantity of palmitoleic acid | 0.011 | SCD |
| Cardiovascular System Development and Function, Cellular Development, Cellular Growth and Proliferation, Connective Tissue Development and Function, Digestive System Development and Function, Hepatic System Development and Function, Organ Development, Organismal Development, Tissue Development | Proliferation of hepatic stellate cells | 0.014 | COL1A1,TIMP1 |
| Carbohydrate Metabolism, Digestive System Development and Function, Hepatic System Development and Function | Glycogenolysis of liver | 0.014 | SCD |
| Energy Production, Lipid Metabolism, Nucleic Acid Metabolism, Small Molecule Biochemistry | Oxidation of palmitoyl-coenzyme A | 0.014 | SCD |
| Cancer, Organismal Injury and Abnormalities | Early stage tumor | 0.018 | FOXM1 |
| Tissue Development | Accumulation of extracellular matrix | 0.021 | COL1A1 |
| Lipid Metabolism, Molecular Transport, Nucleic Acid Metabolism, Small Molecule Biochemistry | Concentration of malonyl-coenzyme A | 0.021 | SCD |
| Lipid Metabolism, Molecular Transport, Small Molecule Biochemistry | Quantity of oleic acid | 0.021 | SCD |
| Cell Death and Survival, Gastrointestinal Disease, Hepatic System Disease, Organismal Injury and Abnormalities | Apoptosis of liver cells | 0.021 | **USP2**,SCD,TIMP1 |
| Cell Morphology | Contraction of hepatic stellate cells | 0.025 | ACTA2 |
| Digestive System Development and Function, Hepatic System Development and Function, Organ Development, Organ Morphology, Organismal Functions | Healing of liver | 0.025 | PLAT |
| Lipid Metabolism, Molecular Transport, Small Molecule Biochemistry | Transport of palmitic acid | 0.025 | FABP5 |
| Cell Death and Survival | Survival of hepatic stellate cells | 0.028 | TIMP1 |
| Lipid Metabolism, Small Molecule Biochemistry | Fatty acid metabolism | 0.031 | SCD,FABP5 |
| DNA Replication, Recombination, and Repair | DNA replication | 0.032 | FOXM1 |
| Carbohydrate Metabolism, Molecular Transport | Depletion of glycogen | 0.032 | SCD |
| Cell Cycle, Hepatic System Development and Function | Mitosis of hepatocytes | 0.032 | FOXM1 |
| Cellular Development, Cellular Growth and Proliferation, Digestive System Development and Function, Hepatic System Development and Function, Organ Development | Proliferation of liver cells | 0.034 | FOXM1,COL1A1,TIMP1 |
| Carbohydrate Metabolism, Lipid Metabolism, Molecular Transport, Small Molecule Biochemistry | Concentration of phosphatidylcholine | 0.035 | SCD |
| Carbohydrate Metabolism, Digestive System Development and Function, Hepatic System Development and Function | Gluconeogenesis of liver | 0.042 | SCD |
| Organismal Injury and Abnormalities, Tissue Morphology | Size of lesion | 0.042 | FOXM1 |
| Digestive System Development and Function, Gastrointestinal Disease, Hepatic System Development and Function, Hepatic System Disease, Organ Morphology, Organismal Development, Organismal Injury and Abnormalities | Abnormal morphology of hepatic sinusoid | 0.045 | FOXM1 |
| Cardiovascular Disease, Gastrointestinal Disease, Hematological Disease, Hepatic System Disease, Organismal Injury and Abnormalities | Budd-Chiari syndrome | 0.049 | PLAT |
| Lipid Metabolism, Molecular Transport, Small Molecule Biochemistry | Concentration of cholesterol ester | 0.049 | SCD |
| Molecular Transport | Transport of molecule | 0.050 | SCD,FABP5 |

Up-regulated molecule is only Usp2 and shown in bold.
